# Towards alternative/complementary wastewater treatment: a review of the recent advancements in photoelectrocatalytic oxidation of sulfonamide antibiotics

**DOI:** 10.1039/d5ra08934d

**Published:** 2026-05-18

**Authors:** Babatunde A. Koiki, Kehinde D. Jayeola, Dimpo S. Sipuka, Tsholofelo I. Sebokolodi, Donggen Chen, Peng Liu, Omotayo A. Arotiba

**Affiliations:** a Department of Chemical Sciences, University of Johannesburg South Africa bakoiki@gmail.com oarotiba@uj.ac.za; b Centre for Nanomaterials Science Research, University of Johannesburg South Africa; c Zhejiang Jinmo Environmental Technology Co., Ltd Zhejiang Shaoxing 312000 China

## Abstract

The occurrence of pharmaceutical compounds in the environment has continued to attract the attention of researchers and this has become the focus of many scientific research work as well as review articles. Sulfonamides have been detected in underground water, seawater, sediment, soil, drinking water, river water, animal manure, treated water effluent and reclaimed water because it is not completely oxidised by most methods used in wastewater treatment plants. Photoelectrocatalytic (PEC) oxidation has distinguished itself as an efficient technology for the abatement of organic pollutants. This review provides insight into recent advancements made in the PEC oxidation of sulfonamides. We carefully captured the progress made in the choice of photocatalysts, light sources, and electrode substrates used. In addition, we also reviewed the recent progress made in the area of heterojunction engineering as this offers a promising and highly efficient approach towards the abatement of sulfonamides. Furthermore, the performances of the commonly known hydroxyl radical PEC and the sulphate radical PEC processes were compared and discussed. An extensive discussion on different types of photoanodes (pristine and modified) and dual photoelectrode systems used for the PEC oxidation of sulfonamides is presented with concluding remarks as well as future perspectives. Overall, this review will furnish the scientific community with insight into highly efficient, cheap, energy-saving, and sustainable technologies to remediate water polluted with emerging pharmaceutical contaminants.

## Abstract 2

PEC oxidation: a promising, efficient, energy saving, and sustainable technology for the removal of sulfonamide antibiotics from wastewater.

## Graphical abstract

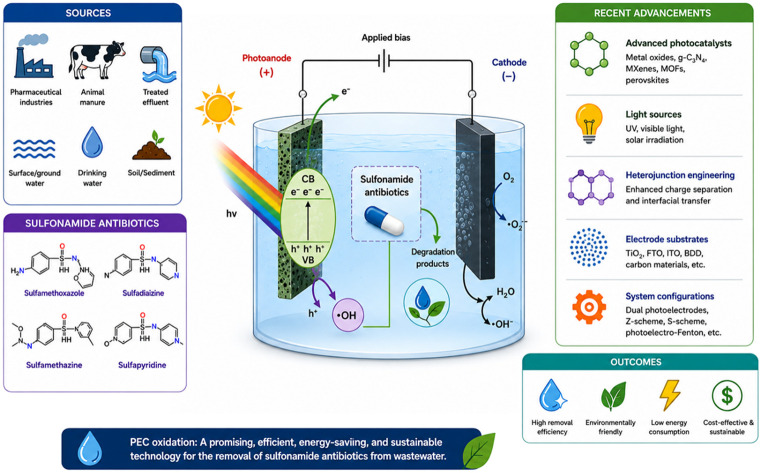

## 1. Introduction

**Fig. 1:**
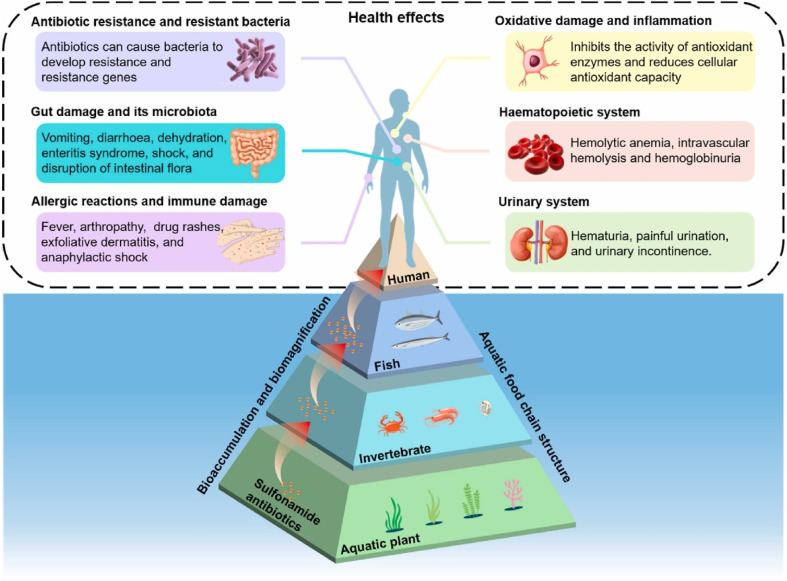
Several health effects of sulfonamide antibiotics, reproduced from ref. 13, with permission from [Elsevier],^13^ copyright 2025.

Antibiotics have been adjudged as an emerging pharmaceutical contaminant in water. When untreated effluents containing some antibiotics are discarded into the environment, they inflict severe damage on human and animal health as well as the ecosystem at large.^1,2^ For over 5 decades, sulphonamides, a group of antibiotics, have been widely applied to stall bacteria from reproducing both in the cells of humans and animals.^3^ In addition, these sulphonamides are the most extensively used antibiotics because they have unique chemical properties and are cheap to produce.^4^ When these drugs are ingested into the body, due to incomplete metabolism, they are not completely absorbed by the bodies of humans and animals, hence they are egested. It has been reported that the egested product contains nearly 75–90% of these drugs in their raw forms when they enter the environment.^5–7^ Owing to incomplete metabolism, these drugs are released into the water system mainly through excretion. For example, about 50 to 100% of the intake dosage of sulfamethoxazole, a sulfonamide, is usually excreted by human beings and animals.^8^ Sulfamethoxazole is mostly detected in wastewater, surface water, soils, and manure.^9,10^ It is a known fact that sulfamethoxazole is persistent and difficult to break down, hence it persists in the environment and to a large extent in surface and groundwater.^2^ Several studies have established the toxic effects of sulfonamide antibiotics on human health and aquatic life^11–13^ and this is summarised in Fig. 1. Therefore, a highly efficient method that can eliminate sulfamethoxazole and other known sulfonamides from the environment must be sought.

Advanced oxidation processes (AOPs) have been regarded as an efficient water treatment technology because of the *in situ* production of strong and highly reactive oxidants, which are non-selective in nature.^14,15^ The complementary effects of oxidants such as hydroxyl radicals, photogenerated holes, and superoxides result in the complete mineralisation of pollutants in water. This caters for the challenge of secondary pollution that is associated with conventional wastewater treatment techniques. Among all the AOPs, photoelectrocatalytic (PEC) oxidation presents itself as a promising green technology with the capacity to effectively degrade emerging contaminants in wastewater.^16^ The utilisation of both sunlight and an external bias potential in PEC gives it an added advantage in performance over AOPs such as electrocatalysis, photocatalysis, Fenton, and photo-Fenton.^17^ This is because, in the presence of semiconductor photocatalysts, the light induces the generation of charge carriers, while the bias potential is responsible for the effective separation of the charge carriers, thus resulting in improved performance of the system.^18^ The PEC system is low cost, clean (green and sustainable), eco-friendly, possesses high oxidising power, versatile (able to treat a myriad of recalcitrant pollutants), and is easy to set up.^19^ Several semiconductor photocatalysts such as TiO_2_,^20^ Cu_2_O,^21^ Bi_2_WO_6_,^22^ and WO_3_,^23^ among many others, have been used in the PEC oxidation of sulfonamides. However, the challenge of recombination of photo-induced charge carriers has hampered the effectiveness of these materials, and thus, research advances are towards the circumvention of these setbacks. Examples of such advancement include doping and the formation of heterojunction. In recent times, the application of a dual photoelectrode PEC system has shown promise as a low-cost, energy-saving, sustainable, and highly efficient technology for treating wastewater containing sulfonamides. No doubt, these strategies produced better results, and there is a need for the scientific community to be furnished with these promising recent advances, hence the rationale behind this review article.

It is important to state that despite the success recorded around photoelectrocatalytic oxidation of sulfonamides and how the technology involved has progressed over the years, no review has been written to furnish the scientific world with the required understanding. While we admit that Prasannamedha and Kumar^24^ in the year 2020, reviewed the contamination of removal of sulfamethoxazole from aqueous solution using techniques such as adsorption, electrochemical oxidation processes, Fenton/photo-Fenton process, photocatalytic process, *etc.* There is no review article that focuses on the PEC approach, which is adjudged to possess some advantages over electrochemical oxidation and photocatalytic processes.^25^ PEC offers a more robust approach in that it combines electrochemical oxidation with photocatalysis. Also, the photocatalyst is immobilised on the electrode in PEC thus mitigating the catalyst recovery challenge. In our opinion, PEC has introduced many dynamics, data, knowledge, and opportunities in the treatment of sulfamethoxazole that are worthy of reporting. Therefore, there is a need for a review article that captures the significant advancement made towards the efficient degradation of sulfonamides which can also serve as a guide towards the treatment of wastewater containing other organic pollutants.

This review presents a critical and in-depth discussion of the recent advancement in photoelectrocatalytic oxidation of sulfonamides. We were able to provide insight into the progress made in the nature and suitability of photocatalysts, light sources, and the choice of electrode substrate in the PEC oxidation of sulfonamides from exfoliated graphite to fluorine-doped tin oxide glass (FTO), nickel foam, and titanium sheet/plate/mesh/foil. Also, we compared the performance of the commonly known hydroxyl radical PEC process with the sulphate radical PEC process (a more recent trend) towards the abatement of sulfonamides. Importantly, we also reviewed the recent advancements made in materials engineering, such as semiconductor heterojunction and dual photoelectrode reactors, as this offers a promising and highly efficient approach towards the abatement of sulfonamides. In addition, the successful applications of different novel photoanodes to decontaminate water containing emerging pharmaceutical pollutants such as these drugs are presented in this review with concluding remarks as well as future perspectives. To the best of our knowledge, no review article has been published that discusses all these recent advancements as contained in this paper. Overall, this review will furnish the scientific community with insight into highly efficient and sustainable technologies to remediate water polluted with emerging pharmaceutical contaminants.

## 2. Sulfonamide antibiotics

### 2.1 Overview

Sulfonamides are antibiotics with a wide spectrum of activity in combating Gram-positive and some Gram-negative bacteria. They were the first antibiotics to be applied in treating human infectious diseases, but are now mostly used for veterinary purposes.^26^ According to the European Union (EU), sulfonamide antibiotics rank second as the most widely applied in veterinary medicine. Statistically, in the UK in 2000, nearly 21% of the drugs sold were sulfonamide antibiotics, while in many other countries in Europe, they accounted for 11–23%. Similarly, in the U.S., sulfonamides constitute 2.3% of all the antibiotics used. The ubiquitous nature of sulfonamide antibiotics spreads even to the African continent as well. For example, in Kenya, they account for 22% of the 14 600 kg of active antimicrobials used in producing animal feeds.^27^ A report focussed on the detection of sulfonamide antibiotics in the aquatic environment between 2016 and 2021 revealed that sulfonamide antibiotics have been detected globally, spanning different continents such as Asia, North America, South America, Europe, and Africa.^28^ The common brands of sulfonamides include the following: sulfamethoxazole, sulfadiazine, sulfacetamide, sulfathiazole, sulfasalazine, sulfadoxine, sulfanilamide, sulfafurazole, and sulfamethizole. They are majorly organo-sulphur compounds consisting of –SO_2_NH_2_ and/or –SO_2_NH groups with unique 5- or 6-membered heterocyclic rings.^29^ The physical properties of some of these sulfonamides, as well as their chemical structures, are presented in Table 1.

**Table 1:** Physical properties of some sulfonamides and their chemical structures

Sulfonamide	Molar mass (g mol^−1^)	Formula	Melting point (°C)	Chemical structure
Sulfamethoxazole	253.28	C_10_H_11_N_3_O_3_S	169	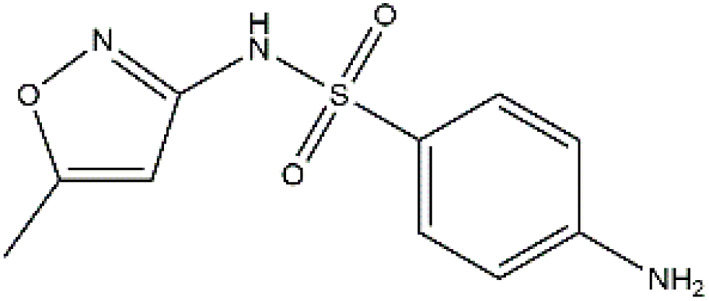
Sulfadiazine	250.28	C_10_H_10_N_4_O_2_S	252–256	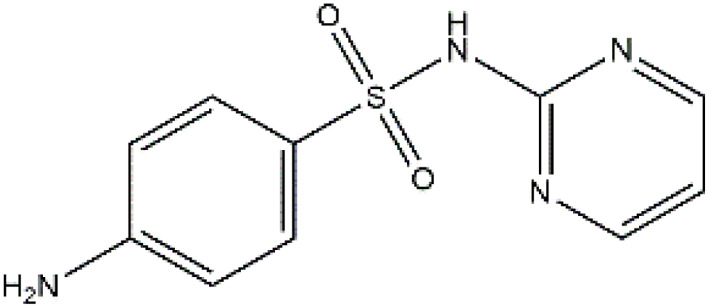
Sulfacetamide	214.24	C_8_H_10_N_2_O_3_S	182–184	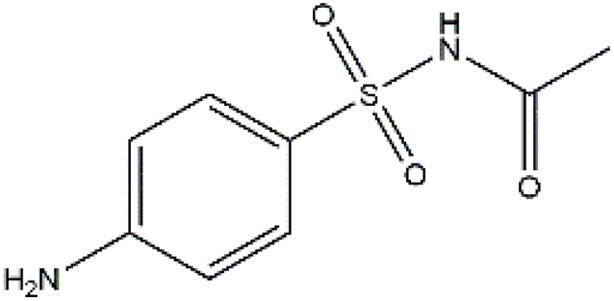
Sulfathiazole	255.31	C_9_H_9_N_3_O_2_S_2_	202–202.5	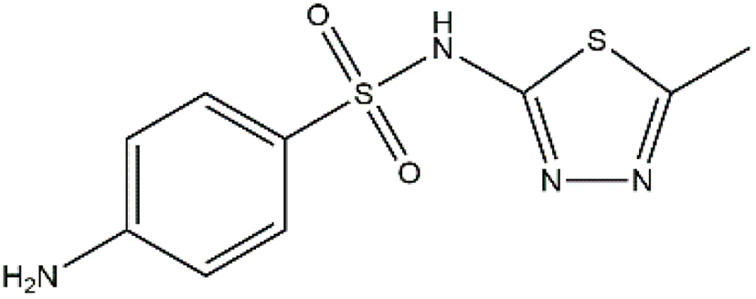
Sulfasalazine	398.39	C_18_H_14_N_4_O_5_S	240–245	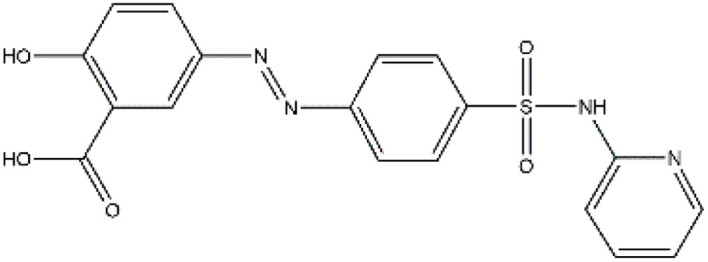
Sulfadoxine	310.33	C_12_H_14_N_4_O_4_S	190–194	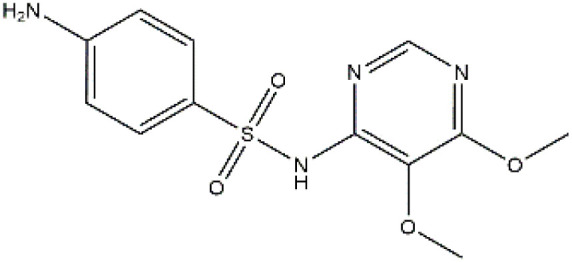
Sulfanilamide	172.20	C_6_H_8_N_2_O_2_S	165	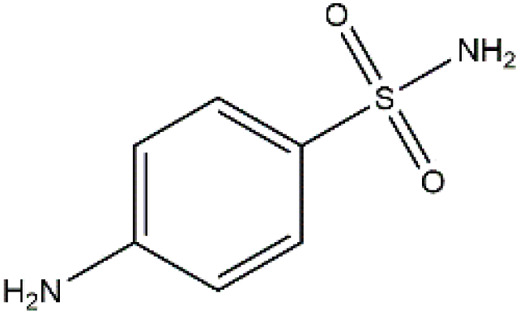
Sulfafurazole	267.30	C_11_H_13_N_3_O_3_S	194	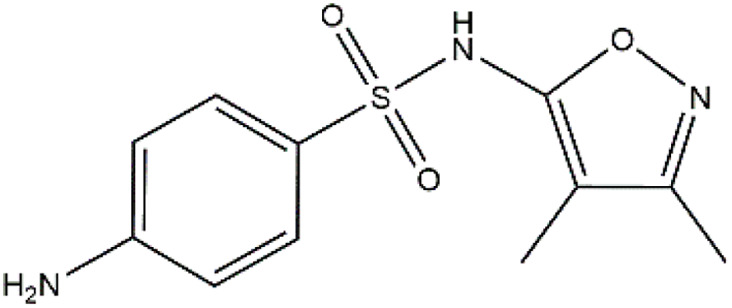
Sulfamethizole	270.33	C_9_H_10_N_4_O_2_S_2_	208	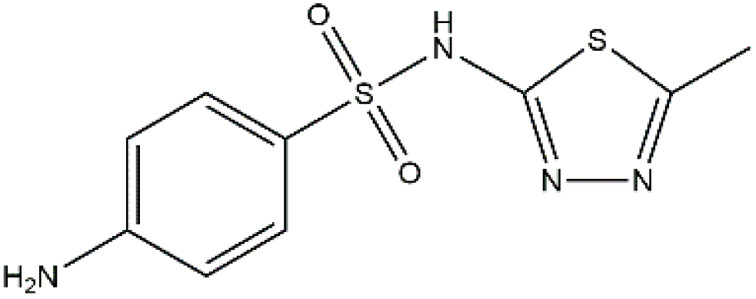

Generally, the process by which sulfonamides are broken down in various organisms takes different routes. For example, when sulfonamides undergo the process of oxidation and acetylation in the body of an organism, this results in the egestion of inactive and reactive metabolites.^12^ According to the European Union directive 93/67/EEC, sulfonamides are classified as harmful or toxic drugs. In addition, in 2009, sulfonamides were classified as highly toxic drugs by Environmentally Classified Pharmaceuticals.^24^ Furthermore, studies have shown that at very low concentrations, sulfonamides pose highly toxic effects on the embryo of zebrafish.^30^ Overall, they are polar and water-soluble compounds with high mobility, hence they have been found in surface water, groundwater, as well as drinking water.^6,31^ Based on the above, it has become imperative to develop efficient wastewater treatment technology for the removal of sulfonamides.

### 2.2 Occurrence of sulfonamide antibiotics in the environment

**Fig. 2:**
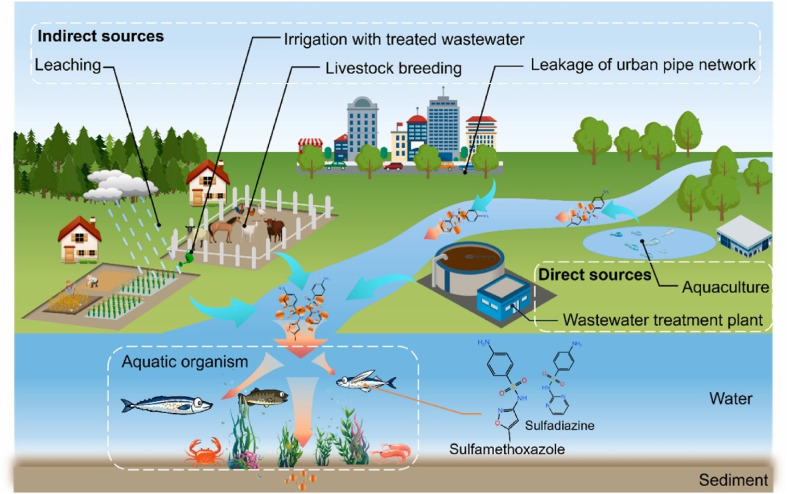
Different routes through which sulfonamide antibiotics enter the aquatic environment, reproduced from ref. 13 with permission from [Elsevier],^13^ copyright 2025.

The occurrence of sulfonamide antibiotics in the environment due to their excessive use has continued to generate a growing concern.^1^ There are two routes through which sulfonamide antibiotics find their way into the aquatic environment: direct and indirect routes as represented in Fig. 2.

Since most effluents arising from wastewater treatment plants are not completely treated, these sulfonamide antibiotics find their way into the ecosystem. For example, a study carried out on the removal rate of some sulfonamide antibiotics in eight urban wastewater treatment plants revealed that 5–62% of sulfamethoxazole, 9–62% of sulfamethazine, 22–68% of sulfapyridine, and 34–79% of sulfadiazine were successfully removed.^32^ This shows the need for a more efficient wastewater treatment technology. In addition, due to the incomplete metabolism of sulfonamide antibiotics in livestock animals, about 70–90% of these drugs are excreted either in their original form or as metabolites.^33,34^ Reports have shown that the detection rate of sulfadiazine and sulfamethoxazole in the Mediterranean Sea is 100%.^35^ Also, sulfonamide antibiotics were found to be the major antibiotics in Chinese lakes in the range of 0–67.18 ng L^−1^.^34^ Some findings have also shown that sulfonamide antibiotics have been detected in drinking water, lakes, rivers, surface water, and groundwater. For instance, 840 ng L^−1^ of sulfadiazine was found in surface water in Kenya;^36^ 1840 ng L^−1^ of sulfamethoxazole was found in groundwater in Taiwan;^37^ sulfamethazine and sulfamethoxazole have been detected in groundwater in about 18 states of the U.S. ranging from 360–110 ng L^−1^.^38^ Furthermore, the amount of sulfamethoxazole in the city canal in Vietnam has reached an elevated concentration of about 4330 ng L^−1^.^39^ In general, the report of Duan *et al.*, confirmed that sulfonamide antibiotics are predominant in the aquatic environment within the concentration range of ng L^−1^ to µg L^−1^.^28^ All these staggering figures inform part of our motivation for selecting sulfonamide as our model antibiotics in this review and to also furnish the scientific community with the recent advances made in the successful treatment of wastewater containing them.

## 3. Wastewater treatment techniques

Conventional water treatment technologies such as coagulation, filtration, flocculation, sedimentation, and others have been reported to be ineffective in remediating water containing sulfonamides.^23^ Also, the possibility of utilising physical methods such as adsorption has been investigated.^40–42^ However, adsorption cannot break down sulfonamides in water but only transfers it from one medium to another leading to secondary pollution. Advanced oxidation processes (AOPs) such as the electrochemical oxidation process,^43^ Fenton/photo-Fenton,^44^ photocatalytic process,^45^ cavitation-based AOPs,^46^ and radiation-enhanced catalytic reaction^47^ have proven to be effective treatment techniques with the ability to breakdown sulfonamides in water, but they suffer some setbacks. For example, electrochemical oxidation methods rely on the use of electrical energy to produce radicals that can degrade pollutants, but this technique is impeded by factors such as electrode lifetime, increased current density, the electrical conductivity of the electrode, as well as the possibility of the electrode undergoing fouling because of the presence of organic deposits on the electrode surface. In addition, the energy consumption rate as well as the low conductance associated with wastewater, which will demand the addition of electrolytes, weakens the application of this technique on an industrial scale.^17,48,49^ Also, the Fenton/photo-Fenton process depends on the production of oxidants from hydrogen peroxide. Although the oxidants generated are capable of degrading sulfonamides, the Fe^2+^ ions undergo precipitation to form Fe(OH)_3_, which is insoluble in nature, thereby leading to secondary waste such as iron-rich sludge.^24,44,50^ Photocatalysis relies on light to produce oxidants to degrade pharmaceutical pollutants, but catalyst recovery after use is laborious, thus making catalyst reusability challenging. In addition, there is rapid recombination of the photogenerated charge carriers which impedes the performance of the catalyst.^51^ The setbacks associated with cavitation-based AOPs include high temperature and pressure, surface destruction and corrosion of the metal surface, while the radiation-enhanced catalytic reaction is energy and frequency-dependent.^24^ Given the above major demerits suffered by all the various techniques discussed, the photoelectrocatalytic oxidation (PEC) process has emerged as a promising technique for the treatment of water containing sulfonamides because PEC is cheap, eco-friendly, and possesses the ability to completely mineralise the pollutant and requires. In addition, it offers high degradation efficiency, low temperature, less energy demand, solar utilization, mild reaction conditions, zero sludge formation, and easy catalyst recovery.^50^

### 3.1 Photoelectrocatalytic oxidation: overview

In addition to other details not specifically mentioned earlier on various AOPs, the PEC process has been identified as an efficient technology in the treatment of water polluted with antibiotics.^52^ This is because of the synergistic effects of both the electrochemical oxidation and photocatalytic degradation processes.^53^ The PEC process is based on the application of bias potential to facilitate the migration of the photogenerated electrons from the conduction band of the photoanodes towards the cathode, thereby giving the photogenerated holes the ease to react directly with the pollutant and degrade them. Also, the photogenerated holes can react directly with water molecules to produce hydroxyl radicals which also complements the degradation process. In addition, the rate of recombination is suppressed due to the application of bias potential, the powdered photocatalyst is easy to recover since they have been immobilised on a substrate, and the recycling efficiency of the photocatalyst is enhanced.^54^ Overall, the PEC process presents itself as a promising and highly efficient technology. PEC has been applied towards the abatement of different emerging pharmaceutical pollutants such as ciprofloxacin, tetracycline, paracetamol/acetaminophen, and diclofenac, among others. Towards the performance of PEC, the choice of materials to be used as photoanodes, the source of light, and the kind of electrode substrate play vital roles.

#### 3.1.1 Nature of materials for photoelectrocatalysis

Light plays a significant role in the PEC process as it promotes the excitation of the photogenerated electrons from the valence band to the conduction band of the semiconductor materials. Therefore, the choice of semiconductor materials is based partly on their light-harvesting properties. Some materials absorb more in the UV region of the electromagnetic spectrum, examples include TiO_2_,^14^ ZnO,^55^ BiPO_4_,^56^*etc.*, while some materials absorb more in the visible spectrum. Since the UV region (100 to 400 nm) accounts for only about 4–5% of the incoming sunlight, the PEC process that depends on UV-active semiconductor materials is not practicable and sustainable.^57^ Furthermore, UV irradiation is unsafe for the environment. As we move towards sustainability and green technology, the search for materials that possess the ability to harvest sunlight (visible light) is warranted.^58^ Several visible light-driven semiconductors have been used as photocatalysts for the treatment of water containing pharmaceutical pollutants. Examples include Cu_2_O, BiVO_4_, Bi_2_WO_6_, Ag_3_PO_4_, WO_3_, Fe_2_O_3_, Bi_2_Sn_2_O_7_, *etc.* In recent years, the solar light-harvesting ability of UV-active semiconductor materials has been promoted by doping,^54^ heterojunction formation,^59^*etc.* Thereby extending their absorption from the UV region into the visible light region.

Since the semiconductor is usually deposited on an electrode/substrate, its conductivity (or electrochemical activity) is also important. For example, the poor conductivity of BiFeO_3_ perovskite was enhanced by the incorporation of graphite nanoparticles (BiFeO_3_/GNPs) which resulted in the marked improvement of the photocurrent response of BiFeO_3_ towards its application in the PEC removal of ciprofloxacin in water.^60^

Resistance to photocorrosion, which is a measure of the stability of the catalyst under prolonged or intense light radiation, is also an important factor. Semiconductors like ZnO fall short in this area and thus the reason for modifying this material. Another factor is the amenability of the semiconductor towards photoelectrode fabrication. Semiconductors that can be easily electrodeposited or that lend themselves to other advanced techniques such as atomic layer vapour deposition can be suitable candidates in photoanode preparation on the premise that the materials pass the test of light harvesting and conductivity.

#### 3.1.2 Light source

The source of light in the PEC process is vital as it contributes to the evaluation of the cost of energy consumption, eco-friendliness, and the toxicity level of the system. The conventional light sources for the PEC oxidation of sulfamethoxazole (and other pharmaceuticals) include UV-A lamps,^50,61^ mercury lamps,^7^ and xenon light.^2^ Recently, researchers have used green light emitting diode (LED) as their light source for the PEC degradation of sulfamethoxazole.^19,54,59,62^ LED possesses some merits over other light sources because it is cheaper, less toxic, less energy consuming, low production of heat, small in size, and possesses a longer lifetime. These merits place LED-based systems at an advantage for future application in the PEC technology for the removal of sulfamethoxazole and other pollutants in general.

#### 3.1.3 Nature of the substrate or the electrode

It is important to present here the significant progress made over the years in the choice of the substrate used in the PEC oxidation of sulfonamides. In all the works captured in this review article, four electrode type have been used in the fabrication of photoanodes towards the degradation of sulfonamides namely exfoliated graphite, FTO glass, nickel foam and titanium sheet/plate/mesh/foil. These electrode types also cut across PEC applications for other priority organic pollutants in water.

The application of exfoliated graphite in electrochemical oxidation and PEC for water treatment was first reported through the earlier works of Arotiba and coworkers.^63–65^ They used EG as a direct oxidation electrode and support for semiconductors towards the oxidation of dyes and phenolic compounds. The application of EG as substrate was extended to the degradation of sulfamethoxazole in a report by Peleyeju *et al.*,^20^ where a TiO_2_-exfoliated graphite electrode was used for PEC degradation of sulfamethoxazole. The only other report was by Mafa *et al.* who prepared a g-C_3_N_4_/BiOI/EG photoanode for the PEC degradation of sulfamethoxazole.^66^ While these two reports are commendable, the challenge is the leaching of the exfoliated graphite into the solution during repeated use of the photoanodes, which weakens the stability and reusability of the electrode. Since the photocatalyst and exfoliated graphite are both compressed into the pellet after physical mixing, the application of bias potential under different current densities causes the exfoliated graphite to gradually leach (partly by oxidation of the carbon) into the solution.^67^ The weakness of EG under high current necessitates a more stable substrate.

Another substrate is fluorine-doped SnO_2_ (FTO), a low resistivity, high conductive and thermally stable glass that allows light to pass through.^68^ FTO glass lends itself as a substrate for many photoelectrode fabrication approaches such as simple casting, electrodeposition,^69^ successive ionic layer adsorption and reaction,^70^ hydrothermal,^23^*etc.* FTO glass has been used as a substrate in fabricating photoanode for the PEC removal of sulfamethoxazole; FTO/Cu_2_O,^21^ FTO/Bi_2_WO_6_,^22^*etc.*

Titanium sheet/plate/mesh/foil is also a versatile material that has been widely applied either for synthesis or as a conducting substrate in PEC water treatment. Titanium plate/sheet/foil has been electrochemically anodised by different researchers to produce TiO_2_ nanotube arrays for PEC oxidation of sulfamethoxazole and sulfadiazine.^59,71–73^ Tailoring titanium into nanotubes and nanostructures has added advantages such as a high surface area to volume ratio, a unique tubular structure that promotes the electron migration efficiency, and improved stability and reusability when compared with the powdered TiO_2_.^74^ As a conducting substrate, Hu and co-workers grew TiO_2_ nanoneedle arrays on titanium mesh *via* hydrothermal synthesis to form a photoanode for the degradation of sulfamethazine.^62^ It is important to state that both the FTO and titanium sheet/plate/mesh/foil can withstand high heat as they can be used to synthesise materials that need to be calcined at elevated temperatures.

Nickel foam is another substrate that has been used in fabricating photoanode for the PEC oxidation of sulfamethoxazole and sulfadiazine. Based on the works captured in this paper, Wu and co-workers were the first and only group to date to report the use of nickel foam as their electrode substrate for PEC oxidation of sulfamethoxazole.^19^ Their motivation for using nickel foam was due to its high electronic conductivity and unique three-dimensional cross-linked lattice structure, resulting in its high surface area, porosity, and adsorption capacity.^75–77^ Also, Wang *et al.* proposed nickel foam as one of the ideal substrates to conduct photocatalysts. This is because, with their 3D mesh, which is responsible for their large surface area, mass transport is promoted during degradation. Also, they are known to be stable and cheap. Motivated by the above, Wang and co-workers doped Fe_2_O_3_ with copper and then grew it on nickel foam for sulfadiazine degradation in a peroxymonosulfate enhanced PEC system.^78^

## 4. Photoelectrocatalytic oxidation of sulfonamides

There has been significant progress made in the PEC oxidation of sulfonamides with several photoanodes. While some of the works entailed the use of single photoanodes, the use of doped photoanodes, the construction of heterojunctions, and the fabrication of dual-photoelectrode systems to enhance the performance of the PEC system are some reported advancements.

### 4.1 Unitary photoanodes

The use of a single or one type of semiconductor material has been reported in the fabrication of photoanodes on substrates such as EG and FTO for the PEC degradation sulfamethoxazole. In their work, Peleyeju *et al.*^20^ prepared (for the first time) a TiO_2_-exfoliated graphite photoanode for the PEC oxidation of sulfamethoxazole. The extent of degradation of sulfamethoxazole was monitored with UV-vis spectrophotometry and they observed a reduction in the absorbance of sulfamethoxazole with time at its *λ*_max_. Also, their study compared the electrochemical and photoelectrocatalytic degradation of sulfamethoxazole at the TiO_2_-exfoliated graphite electrode. The result obtained confirmed that the PEC process performed better than the electrochemical oxidation process. The better performance is because of the complementary effects of the bias potential and solar energy in the PEC system which resulted in the generation of oxidants such as photogenerated holes and hydroxyl radicals which freely reacted with the sulfamethoxazole until it was completely broken down. Furthermore, the study gave insight into the role of the exfoliated graphite in the TiO_2_-exfoliated graphite photoanode. It was reported that the TiO_2_-exfoliated graphite photoanode performed better as compared to only exfoliated graphite. This is because of the following: (i) the TiO_2_-exfoliated graphite has a higher electro-active surface area, thereby resulting in an increased number of sites for the water molecules to be oxidized to produce hydroxyl radicals, (ii) since upon irradiation, photogenerated charge carriers are produced, the exfoliated graphite serves as an electron sink, thereby reducing the rate of recombination. This study further showed that increasing the current density promotes the generation of oxidants at the anode as well as the migration of the electrons towards the cathode. While this work provides insight into the use of exfoliated graphite as support, Peleyeju *et al.* did not probe the stability and reusability of the electrode.^20^ Furthermore, the performance of the system in a real matrix cannot be inferred because the authors did not use real wastewater. The degradation time of 6 h for 100% degradation of sulfamethoxazole (from UV/vis spectrometer) has now been shortened in recent trends.

Cu_2_O has been used as a unitary photoanode material for the PEC degradation of sulfamethoxazole.^79^ Koiki *et al.* used Cu_2_O to generate sulphate radicals for the degradation of sulfamethoxazole.^21^ Although PEC degradation of sulfamethoxazole using hydroxyl radicals has been widely reported, but due to stronger oxidising ability, higher selectivity, longer lifetime, and its ability to break down stubborn organics, sulphate radical PEC degradation technology presents itself as a more efficient technique.^80^ The need to harness the sulphate radical PEC towards improved degradation of sulfamethoxazole led Koiki *et al.* to use a visible-light-driven semiconductor photocatalyst, pristine Cu_2_O to activate persulphate ions to produce sulphate radicals. Cu_2_O was synthesised *via* a rapid and template-free route and then conducted on FTO glass. This study provided a better understanding and insight into the fact that when Cu_2_O is irradiated, electrons and holes are generated (eqn (1)). The electrons in the CB get trapped by the persulphate ions to produce sulphate radicals (eqn (2)). Similarly, while the photogenerated holes in the VB react directly with the sulfamethoxazole to break them down (eqn (3)), it also reacts with the water molecules to produce hydroxyl radicals (eqn (4)).

1 Cu_2_O + *hv* → h^+^ + e^−^

2 e^−^ + S_2_O_8_^2−^ → SO_4_˙^−^ + SO_4_^2−^

3 h^+^ + sulfamethoxazole → CO_2_ + H_2_O

4 h^+^ + H_2_O → ˙OH + H^+^

Given the complementary effects of sulphate radicals, hydroxyl radicals, and holes, 84% of sulfamethoxazole was oxidised within 2 h. In addition, the trapping of the photogenerated electrons by the persulphate ions assisted in suppressing the rate of recombination commonly associated with PEC degradation experiments using pristine photoanodes. The reusability and stability of the electrode were probed by conducting cycles of experiments, and it was found to be relatively stable and reusable after 10 hours. Towards real-life application, the authors probed the efficiency of their material in a real wastewater system resulting in a 50% mineralisation from total organic carbon measurement after 2 h.^21^ Compared to results obtained from Peleyeju *et al.* as earlier discussed,^20^ it is clear that there has been advancement in the PEC oxidation of sulfamethoxazole using pristine photoanodes. Also, the issue of electrode reusability and stability, treatment duration and real-life practicability has been addressed. Therefore, this study offers a more efficient, reusable, and stable approach towards the PEC oxidation of sulfamethoxazole using pristine photoanodes.

### 4.2 Doped photoanodes

To overcome the problem of rapid recombination that is generally associated with the use of single material (or pristine) photoanodes in the PEC oxidation process, researchers in recent studies have explored the possibility of introducing dopants into these semiconductor photocatalysts. It is expected that in the presence of dopants, the rapid recombination of the photogenerated charge carriers is suppressed, and the solar light harvesting properties of the photoanodes are improved. Thus, the overall performance of the system is enhanced.

ZnO possesses a wide band gap, and it is therefore only active in the UV region of the electromagnetic spectrum. However, Yeganeh *et al.* showed that by doping ZnO with cobalt, (i) the absorption of ZnO can be extended towards visible light, (ii) the band gap energy can be altered to promote electron transfer, (iii) the Fermi level of ZnO can be tuned, and (iv) the overall PEC performance of the system can be enhanced. Therefore, in their work, cobalt-doped ZnO was depositedon an FTO glass and the photoanode was applied for the photoelectrocatalytic oxidation of four sulfonamide antibiotics (sulfacetamide, sulfathiazole, sulfamethoxazole, and sulfadiazine) in the presence of visible light. This study further provides insight into the performance of the synthesised photoanode under different systems such as photolysis (PL), electrochemical oxidation (EC), photocatalysis (PC), and photoelectrocatalytic degradation (PEC). The results obtained showed that for the sulfamethoxazole, PL, EC, PC, and PEC yielded 10.6, 27.3, 50.2 and 95.8% degradation respectively. This marked performance by the PEC confirmed that the complementary effect arising from the EC and PC enhanced the degradation of sulfamethoxazole. This is due to the suppression in the recombination of the photogenerated charge carriers because of doping ZnO with Co. This further gave rise to the production of more oxidants such as hydroxyl radicals, holes, and superoxides, which contributed to the degradation process.^7^

**Fig. 3:**
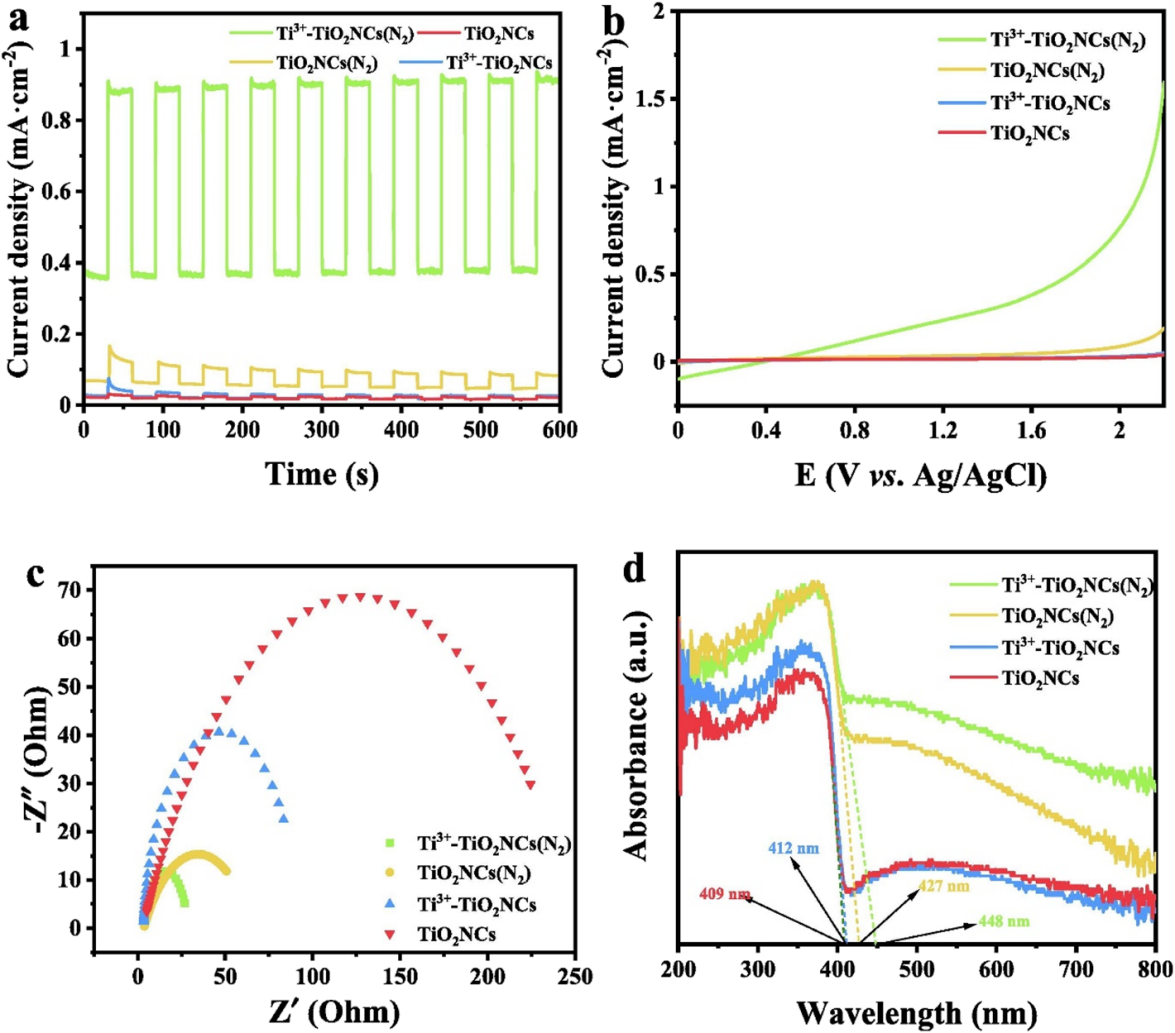
(a) Variation of photocurrent response under the light on/off conditions, (b) linear sweep voltammetry under visible light irradiation, (c) EIS Nyquist plot under visible light irradiation, and (d) UV-vis absorbance spectra of Ti^3+^–TiO_2_ NCs(N_2_), TiO_2_NCs(N_2_), Ti^3+^–TiO_2_ NCs, and TiO_2_ NCs, reproduced from ref. 54, with permission from [Elsevier],^54^ copyright 2023.

TiO_2_ is one of the most widely used photoanodes in the PEC oxidation of antibiotics because it is cheap, possesses excellent chemical stability, and has interesting photoelectrochemical properties. Nevertheless, it possesses a wide bandgap, poor solar light harvesting properties, and suffers from rapid recombination. While the conventional metal doping method has been used to mitigate these setbacks, it further imparts negatively on the thermal stability as well as the photocatalytic properties of the material.^81,82^ On the other hand, self-doping has been identified as a preferable method because it prevents the introduction of other elements and induces intrinsic defects (Ti^3+^ or O_v_ (oxygen vacancy)) in the TiO_2_ crystals. Interestingly, the Ti^3+^ increases the electrical conductivity and promotes the charge carrier separation. In addition, the Ti^3+^ or O_v_ can also introduce new defect energy levels ranging between 0.75–1.18 eV below the CB of TiO_2,_ thereby enhancing its visible light activity. Furthermore, it has been established that constructing specific nanoarchitectures can also effectively suppress the rate of recombination by shortening the distance it will take the minority charge carriers to get to the surface. The above motivated Liang *et al.* to construct an efficient Ti^3+^ self-doped and nitrogen-annealed TiO_2_ nanocone arrays Ti^3+^–TiO_2_ NCs(N_2_) photoanode for the PEC abatement of sulfamethazine.^54^ The separation of the photogenerated charge carriers was studied (Fig. 3). As shown in Fig. 3a, the photocurrent response obtained by the Ti^3+^–TiO_2_ NCs(N_2_) photoanode was about 9.5 times higher than pristine TiO_2_ NCs(N_2_). The linear sweep showed a similar photocurrent pattern (Fig. 3b) this marked increase was due to improved photogenerated charge carrier separation caused by the introduction of Ti^3+^(self-doping). Furthermore, the EIS Nyquist plot (Fig. 3c) showed that the Ti^3+^–TiO_2_ NCs(N_2_) photoanode possessed the least *R*_ct_ value given the size of the semicircle. This showed that the charge transfer kinetics taking place at the interface between the Ti^3+^–TiO_2_ NCs(N_2_) photoanode and the electrolyte significantly suppressed the recombination of the electrons and holes. The results obtained from the UV-vis diffuse reflectance spectroscopy (Fig. 3d) showed that annealing the TiO_2_ NCs in nitrogen as well as the introduction of Ti^3+^ caused the photoanode to absorb more in the visible light and a reduced band gap energy. Liang and co-workers went ahead to interrogate the PEC performance of the prepared electrodes by applying them towards the abatement of sulfamethazine. Expectedly, the pristine TiO_2_NCs photoanode degraded 47.17% of sulfamethazine, while the Ti^3+^–TiO_2_ NCs(N_2_) photoanode degraded 98.69% within 60 min confirming that the separation of the photogenerated charge carriers has significantly been promoted in the doped photoanode. Overall, this work offers a promising approach for treating effluents containing pharmaceuticals by applying Ti^3+^ self-doped and nitrogen-annealed TiO_2_ nanocone arrays.^54^

Towards photocatalyst improvement, another approach to doping is defect engineering.^83–86^ Defect engineering entails tuning the electrical structure of pristine semiconductor materials. One of the methods in defect engineering is to introduce oxygen vacancies (O_V_) to significantly improve the reactivity of the active sites, thus markedly boosting the PEC performance of the photocatalyst.^87,88^ The possibility of exploring this promising approach became the motivation for which Wu *et al.* developed a direct approach that promoted a concomitant boron doping and O_v_ production on bismuth tin oxide (Bi_2_Sn_2_O_7_) quantum dots for PEC oxidation of sulfamethazine.^19^ The results obtained from the optical and photoelectrochemical studies showed that the synergistic effects of boron doping and O_v_ gave rise to the following: (i) the band gap of the pristine Bi_2_Sn_2_O_7_ became narrower after doping, thus improving the solar light harvesting property of Bi_2_Sn_2_O_7_ and enhancing its performance towards the PEC oxidation of sulfamethazine, and (ii) the current density of 20% boron-doped Bi_2_Sn_2_O_7_ was nearly twice higher than the pure Bi_2_Sn_2_O_7_ showing that doping with boron resulted in the efficient separation of the charge carriers. It is important to note that the effect of boron doping, as well as O_v_ on the electronic structure of Bi_2_Sn_2_O_7_ was also investigated *via* Density Functional Theory. The results obtained from the calculations confirmed that the boron doping and the introduction of O_v_ on the Bi_2_Sn_2_O_7_ successfully tuned the electronic structure of Bi_2_Sn_2_O_7_ by creating intermediate levels. These intermediate levels promoted excitation, improved charge separation and electron transport, hence suppressing the recombination of the photogenerated electron and holes. Finally, the efficiency of the photoanodes was probed in a PEC degradation experiment to break down sulfamethazine. As a result of the rapid recombination and poor light-harvesting property of Bi_2_Sn_2_O_7_, only 48.4% of sulfamethazine was broken down within 60 min. Conversely, 20% boron-doped Bi_2_Sn_2_O_7_ completely degraded sulfamethazine under 60 min. Conclusively, this work offers a new approach to the collective effect arising from boron doping and O_v_ in heterobimetallic oxides such as Bi_2_Sn_2_O_7_ in a PEC system. Furthermore, given the efficiency of the photoanode in completely degrading sulfamethazine within an hour under an LED light source, this work presents a novel, cheap, eco-friendly, energy-saving, and efficient technology for water treatment.

A recent methodology for preparing doped semiconductor photoanodes for PEC oxidation is through the use of materials based on metal–organic frameworks (MOFs). MOFs are known to possess large surface area, porous, possess high adsorption capacity, and their pore sizes can be tuned.^2^ For example, zeolite imidazole framework (ZIF) is an MOF that contains carbon and nitrogen-rich imidazole which can act as a ligand for metal–organic linkage. Several ZIF-based photocatalysts have been fabricated into electrodes for PEC application.^89^ However, ZIF suffers some setbacks such as poor graphitic structure and as such does not promote electron transport. To circumvent this challenge, Thamilselvan *et al.*^2^ prepared a Ni–Co bimetallic decorated dodecahedral ZIF for the PEC oxidation of sulfamethoxazole. It was proposed that doping the ZIF with Ni and Co will improve the surface area and the current density of ZIF, thereby resulting in improved charge transfer efficiency, and overall, enhanced PEC performance. Rather than generating the usual OH radical for the degradation, the authors prepared Ni–Co bimetallic ZIF to activate peroxymonosulphate (PMS) salt to produce sulphate radicals which will complement other oxidants in the system for enhanced PEC oxidation of sulfamethoxazole. The author justified the effect of doping of the ZIF by the improved PEC degradation in comparison to ZIF only based on the following: (i) doping of ZIF with Ni and Co increased the pore volume as well as the micro-mesoporous structure of ZIF. This further markedly improved the transfer of electrons between PMS and Ni–Co ZIF which led to the production of sulphate radicals, (ii) the Ni–Co ZIF composite photoanode possessed a large surface area, thereby providing more active sites that in turn improved the PEC performance, (iii) the micropores and mesopores of the Ni–Co ZIF composite were able to trap visible light which then induced the production of charge carries such as holes for improved PEC degradation of sulfamethoxazole. Furthermore, they showed that the Ni–Co bimetallic ZIF activated the PMS based on the marked improvement in degradation in the presence of PMS over its absence. With a high degradation efficiency in 24 min, the work opens new possibilities in the development of porous bimetallic/MOFs heterostructured photoanodes for PMS activation for enhanced PEC oxidation of sulfamethoxazole.

### 4.3 Heterostructured photoanodes

**Fig. 4:**
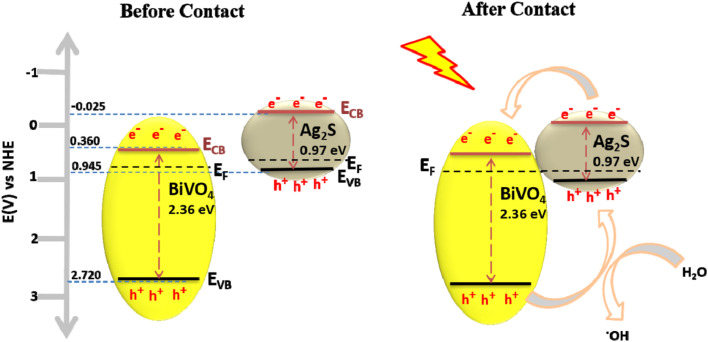
Band alignment between BiVO_4_ and Ag_2_S, reproduced from ref. 90 with permission from [Springer Nature],^90^ copyright 2020.

The formation of semiconductor heterojunctions is a proven effective approaches to PEC oxidation of sulfonamides owing to improved solar light harvesting ability and improved photogenerated charge separation. These lead to increased lifespan of the photogenerated charge carriers, thus making the PEC degradation system more effective. Factors such as band gap, valence band edge, conduction band edge, work function, and Fermi level of the material (semiconductor) are considered in heterojunction engineering. Several heterostructured photoanodes consisting of p–n, n–n, arranged in different configurations such as Z or S scheme heterojunctions, have been developed towards improved PEC oxidation of sulfonamides. For example, Orimolade *et al.*^90^ prepared a p–n heterojunction photoanode consisting of BiVO_4_ and Ag_2_S *via* electrodeposition and successive ionic layer adsorption and reaction towards PEC oxidation of sulfamethoxazole. As shown in Fig. 4, the formation of a p–n heterojunction brought about an alignment of the Fermi energy level, thus creating an internal electric field that resulted in the effective separation of the holes into the VB of Ag_2_S, thereby leading to a direct oxidation of the sulfamethoxazole by the holes. On the other hand, since the electrons moved towards the CB of BiVO_4_, there was efficient charge separation which gave the holes the freedom to further react with water molecules to produce hydroxyl radicals, which also oxidised the sulfamethoxazole. Overall, 86% of the sulfamethoxazole was oxidised within 120 min.

In another work, Fan *et al.*^72^ reported an n–n nano-heterojunction photoanode consisting of highly oriented and vertically ordered stoichiometric copper and zinc-based ferrites (Cu_0.5_Zn_0.5_Fe_2_O_4_) quantum dots anchored with TiO_2_ nanotube arrays (NTs). The TiO_2_ nanotube arrays was first prepared by electrochemical anodization after which it was anchored with Cu_0.5_Zn_0.5_Fe_2_O_4_*via* a noel vacuum-assisted impregnation approach. The results obtained showed that the overall performance of 70% PEC degradation obtained by the heterostructured photoanode can be attributed to the following: (i) improved visible light harvesting property, (ii) effective charge separation arising from the construction of the n–n heterojunction which greatly retarded the rate of recombination. It is important to also note that in this work transient absorption spectroscopy was used to quantitatively estimate the lifetime of the charge carriers. From the results obtained, the holes in Cu_0.5_Zn_0.5_Fe_2_O_4_ quantum dots anchored with TiO_2_ nanotube arrays possessed a longer lifetime of 72.23 µs compared to pristine TiO_2_ NTs (51.49 µs). This also confirmed that the anchoring TiO_2_ NTs with Cu_0.5_Zn_0.5_Fe_2_O_4_ quantum dots extended the lifetime of the photogenerated holes thereby resulting in improved performance of the system. In conclusion, this study has further enriched the scientific community with novel insight into molecular tailing which entails the anchoring of TiO_2_ NTs with multiple spinels such as Cu_0.5_Zn_0.5_Fe_2_O_4_ quantum dots to form a photoanode that is highly efficient, stable, and recyclable for PEC degradation of emerging pharmaceutical pollutants in the presence of sunlight.

To mitigate the challenges of wide band gap and the rapid electron–hole recombination that impede the performance of highly ordered and vertically oriented TiO_2_ NTAs, Teng and co-workers engineered Ag_3_PO_4_/MoS_2_/TiO_2_ NTAs p–n–n heterojunction photoanode and further applied it towards the degradation of sulfadiazine.^91^ Firstly, the TiO_2_ NTAs were prepared by the anodic oxidation method. Thereafter, MoS_2_ was deposited on the as-prepared TiO_2_ NTAs *via* a light-assisted electrochemical deposition approach. Finally, A_3_PO_4_ was conducted on the MoS_2_/TiO_2_ NTAs by successive chemical method. The results from photoelectrochemical degradation studies showed that approximately 70% of the sulfadiazine was degraded by the Ag_3_PO_4_/MoS_2_/TiO_2_ NTAs heterojunction photoanode within 4 h compared to 35% and 44% extent of degradation recorded using MoS_2_/TiO_2_ NTAs and Ag_3_PO_4_/MoS_2_/TiO_2_ NTAs respectively. This performance by the ternary photoanode can be attributed to the antenna effect that can occur in photocatalytic systems. The antenna effect describes what happens when the analyte of interest adheres to the surface of the semiconductor photocatalysts at a certain distance from the light-absorbing particle. Provided that the semiconductor photocatalysts are in an aggregated form and possess the same crystallographic orientation, the light-absorbing particle will transfer energy from one photocatalyst to another. As this energy reaches the photocatalyst that the analyte of interest has been adhered to, the latter then traps the holes, and this causes the separation of the original excitons which overall improves the performance of the system.^91,92^

**Fig. 5:**
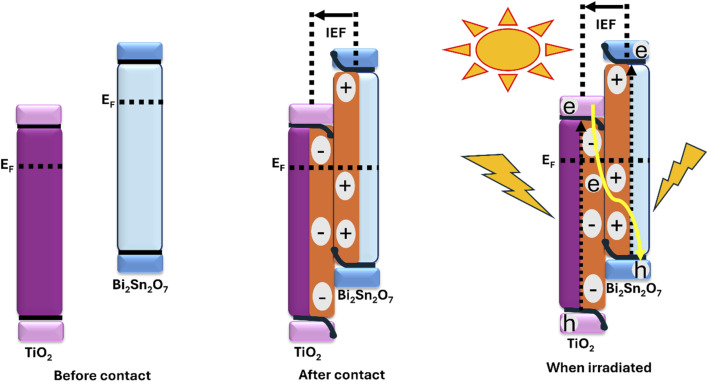
S-scheme heterojunction formation of Bi_2_Sn_2_O_7_/TiO_2_ NTAs: separation of the charge carriers, and the PEC degradation of sulfamethazine.

The construction of S-scheme heterojunction is a novel and more efficient route to heterojunction engineering owing to its unique internal electric field (IEF) effect, which results in a more improved charge carrier separation and greater redox power when compared to type-II and Z-scheme heterojunctions.^93^ Wu *et al.* constructed a Bi_2_Sn_2_O_7_ quantum dots/TiO_2_ nanotube arrays (NTAs) S-scheme heterojunction for improved PEC oxidation of sulfamethazine.^59^ The Bi_2_Sn_2_O_7_ quantum dots were decorated on the TiO_2_ NTAs *in situ via* a hydrothermal process followed by extensive characterisation. The *ex situ* spectra obtained from the XPS showed that after decorating the Bi_2_Sn_2_O_7_ quantum dots on TiO_2_ NTAs, the binding energy of Bi 4f, Sn 3d, and O 1s in Bi_2_Sn_2_O_7_ had the tendency to move to a higher direction, while the Ti 2p and O 1s in TiO_2_ could shift to a lower direction. From these shifts and based on the different work functions of the two materials, there is a possibility of transport/transfer of electrons from Bi_2_Sn_2_O_7_ to TiO_2_ after contact, thus creating an internal electric field effect. To corroborate this, the *in situ* irradiation XPS studies of Bi_2_Sn_2_O_7_ quantum dots/TiO_2_ nanotube arrays revealed that there were shifts in the binding energy of the different core-level orbitals of the materials both in the presence and absence of light. These changes of the core-level orbitals of the materials evidence the electron transfer pathway from TiO_2_ to Bi_2_Sn_2_O_7_ when irradiated, which agrees with the S-scheme mechanism. To gain further insights into the transfer of electrons in the engineering of semiconductor heterojunctions, the work function is a critical parameter. In this work, the results obtained from the work functions of Bi_2_Sn_2_O_7_ quantum dots and TiO_2_ nanotube arrays showed that the Fermi level of TiO_2_ was lower than that of Bi_2_Sn_2_O_7_. However, when these two are in contact, electrons are expected to flow from Bi_2_Sn_2_O_7_ to TiO_2_, promoting an equal Fermi level between the two materials and further resulting in the creation of an internal electric field at the Bi_2_Sn_2_O_7_/TiO_2_ nanotube arrays interface. Overall, this will promote the separation of the charge carriers as well as improve the PEC efficiency towards the degradation of sulfamethazine. As shown in Fig. 5, the engineering of S scheme configuration (Bi_2_Sn_2_O_7_/TiO_2_ NTAs photoanodes) gave room for the photogenerated holes in the valence band of TiO_2_ and the electrons in the counter electrode to be actively involved in the degradation process as this resulted in 90% PEC degradation of sulfamethazine as against a degradation of 64% with pristine the pristine TiO_2_ NTAs photoanode. Furthermore, the Bi_2_Sn_2_O_7_/TiO_2_ photoanode was used in a comparative study to probe into the effectiveness of EC, PC, and PEC processes towards sulfamethazine degradation. The PEC markedly outperformed EC and PC. Overall, the results from this work showed an advancement in the oxidation of sulfamethazine through the fabrication of highly efficient novel heterojunction photoanode and it also provides insight into novel heterojunction engineering that will pave the way for a more efficient wastewater treatment system.

### 4.4 Dual photoelectrodes

As the quest towards sustainable, cheap, highly efficient, and low energy-demanding PEC technologies continues, a dual photoelectrode cell lends itself as a promising approach. This is because it caters for setbacks such as high cost, high energy consumption (1.0–2.0 V *vs.* Ag/AgCl), corrosion of photoanode or photocathode materials, loss in material integrity through self-redox reaction *etc.*^94^ In a dual photoelectrode system, the photoanode and the photocathode serve as light absorbers, and the difference between the quasi-Fermi energy levels of these two photoelectrodes produces an internal photovoltage.^95^ This internal photovoltage can make up for part or the total required electrical energy for the PEC process.^96^

**Fig. 6:**
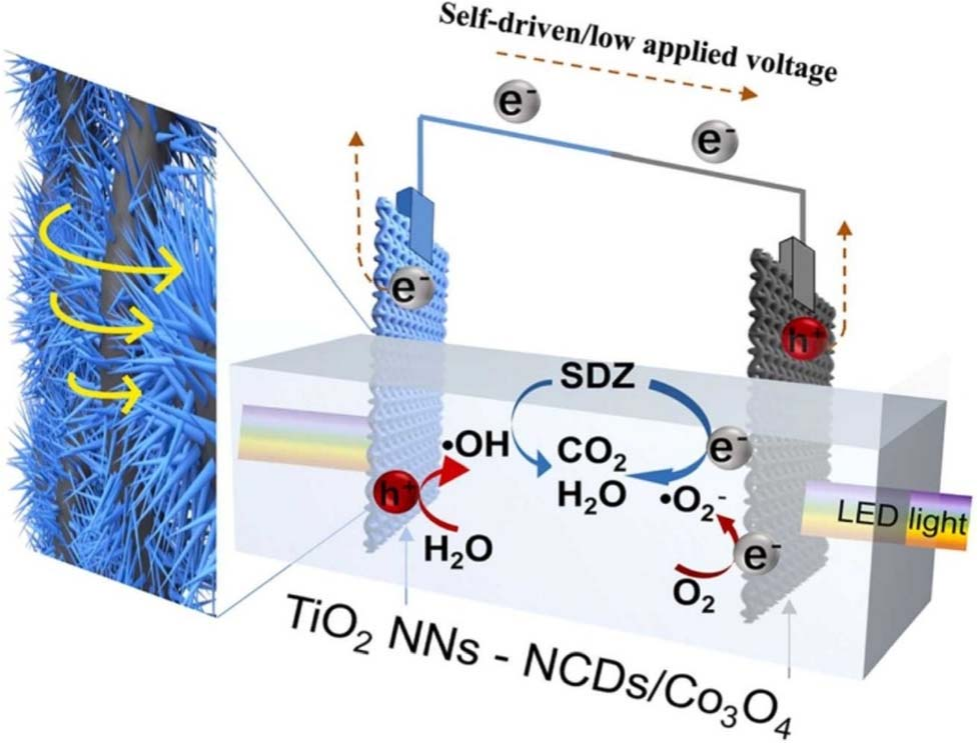
Schematic representation of sulfadiazine degradation by TiO_2_ NNs-NCDs/Co_3_O_4_ PEC system, reproduced from ref. 97 with permission from [Elsevier],^97^ copyright 2023.

Hu *et al.* designed a novel tandem PEC reactor to work at a low/no applied voltage.^97^ This reactor consists of TiO_2_ nanoneedle arrays (NNs) as a photoanode and a nitrogen-doped carbon dots (NCDs) modified Co_3_O_4_ photocathode. A three-dimensional Ti mesh was used as the electrode substrate to promote mass transfer (see scheme in Fig. 6). In their work, the performance of the reactor was investigated by applying it for PEC degradation of sulfadiazine. The results obtained showed that the TiO_2_ NNs/Ti mesh photoanode and the NCDs/Co_3_O_4_/Ti mesh photocathode system achieved 98.54% sulfadiazine degradation within 75 min. This performance can be attributed to the following: (i) the difference between the quasi-Fermi energy levels of these two photoelectrodes produced an internal photovoltage. (ii) The nitrogen-doped carbon dots present in the NCDs/Co_3_O_4_/Ti mesh photocathode served as an electron sink, thus preventing the electrons from reacting with the Co_3_O_4_. The electrons captured by the nitrogen-doped carbon dots reacted with dissolved oxygen, thus producing superoxides which resulted in the reduction of sulfadiazine. (iii) The bias potential introduced into the system (0.4 V *vs.* Ag/AgCl) promoted the further separation of the photogenerated electron and holes thereby creating mobility of the charge carriers to independently take part in the degradation of sulfadiazine. Furthermore, this system shows more promise in practical application as 95.91% of sulfadiazine was degraded within 300 min in the presence of sunlight and absence of external voltage, while 98.16% of sulfadiazine was degraded within 180 min in the presence of sunlight and 0.4 V *vs.* Ag/AgCl. Overall, Hu *et al.* provided a bedrock for further developments of low-cost, energy-saving, efficient, and sustainable PEC technology for wastewater remediation.

The architecture of a dual-function Z-scheme heterojunction PEC system for the degradation of sulfadiazine was fabricated by Leng *et al.*.^98^ In their work, they harnessed the advantages of the band structures of both the photoanode (Ag–TiO_2_/Ti) and the photocathode (ZnO/Cu_2_O/Cu) in the presence of an external bias potential to boost the oxidation and reduction efficiencies of the anode and cathode respectively. When irradiated, the n-type Ag–TiO_2_/Ti and p-type ZnO/Cu_2_O/Cu produced electrons and holes. The introduction of the bias potential aided the movement of electrons from the anode to the cathode, while the holes moved from the cathode towards the cathode. Since these electrodes align, the following occurred: (i) both the photoanode and the photocathode compensated each other with photovoltage, which then enhanced the upward bending of the energy bands of the Ag–TiO_2_/Ti and the downward bending of the ZnO/Cu_2_O/Cu. (ii) The space charge layer thickened. This aided the charge carrier separation and intensified the oxidation reaction on the Ag–TiO_2_/Ti electrode as well as the reduction reaction on the ZnO/Cu_2_O/Cu electrode. The performance of the system was evaluated and 89.1% sulfadiazine degradation within 2 h. Overall, this system offers a novel, stable, and low-energy-consuming approach to wastewater treatment.

## 5. Possible photoelectrocatalytic oxidation pathways of sulfonamides

It is important to state that despite the varied approaches employed by different researchers towards the efficient PEC degradation of sulfonamides, comparative analysis can be drawn from the chemical reactions, proposed degradation pathways, and intermediate products obtained based on the analyte of interest.

### 5.1 Chemical reactions, proposed degradation pathways, and intermediate products involved in the PEC degradation of sulfamethazine

**Fig. 7:**
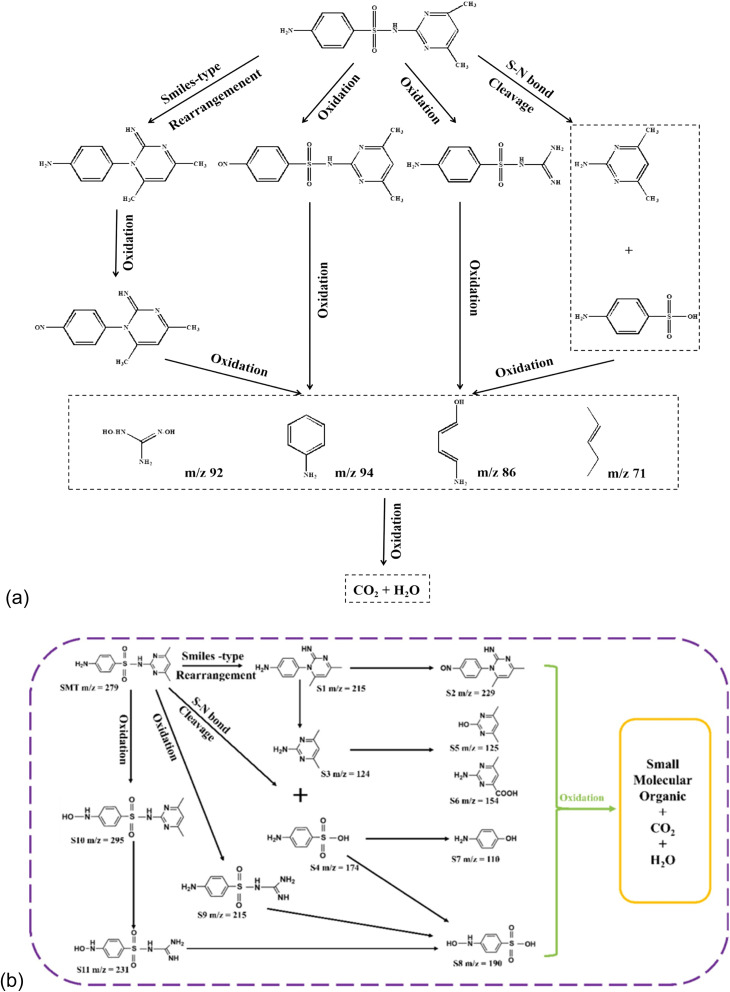
(a) Possible pathway for sulfamethazine degradation, reproduced from ref. 19 with permission from [Elsevier],^19^ copyright 2023. (b) Possible degradation pathway of sulfamethazine degradation, reproduced from ref. 54 with permission from [Elsevier],^54^ copyright 2023.

As shown in Fig. 7a and b,^19,54^ there are four possible chemical reactions and degradation pathways associated with the degradation of sulfamethazine.

#### 5.1.1 Ring rearrangement

The parent compound with *m*/*z* = 279 can undergo a Smiles-type rearrangement to produce 4-(2-imino-4,6-dimethylpyrimidin-1(2*H*)-yl) aniline with *m*/*z* = 215, releasing SO_2_. This is always followed by an oxidation reaction where the nitrogen atom in the amino group of the aromatic ring is converted into a nitroso derivative with *m*/*z* = 229.^99,100^

#### 5.1.2 Bond cleavage

The cleavage of the sulforaphane (S–N) bond, which is between the benzene and pyrimidine ring, can lead to intermediate products such as sulfanilic acid with *m*/*z* = 172 and 2-amino-4,6-dimethylpyrimidine with *m*/*z* = 124. The methyl group of 2-amino-4,6-dimethylpyrimidine can undergo rapid oxidation. This oxidation is initiated by the reactive oxygen species generated by the PEC system to form another intermediate product with *m*/*z* = 154. Also, a substitution reaction occurs at this stage, where the amino groups are substituted by the reactive oxidants, such as OH, to form another intermediate product with *m*/*z* = 125. The sulfanilic acid can also undergo oxidation to give two other products with *m*/*z* = 110 and 190.^100,101^

#### 5.1.3 Ring opening

This is initiated by the hydroxyl radicals and/or other reactive species produced in the PEC system. Here, the C=N and C–N bonds present in the pyrimidine ring of sulfamethazine are affected, leading to ring opening and subsequent formation of 4-amino-ncarbamimidoyl-benzenesulfonamide with *m*/*z* = 215. Further cleavage of the N–S bond of the 4-amino-ncarbamimidoyl-benzenesulfonamide gives rise to another intermediate product with the *m*/*z* = 190.^102^

#### 5.1.4 Hydroxylation

Due to the pronounced effect of the negative charge existing between the N-atom and the benzene ring, the N–H became susceptible to oxidation by the reactive oxygen species, and 4-NO-sulfamethazine is formed with *m*/*z* = 295. This intermediate product further undergoes ring-opening and bond–cleavage reactions to form intermediates with *m*/*z* = 231 and 190, respectively.^99,101^

### 5.2 Chemical reactions, proposed degradation pathways, and intermediate products involved in the PEC degradation of sulfamethoxazole

**Fig. 8:**
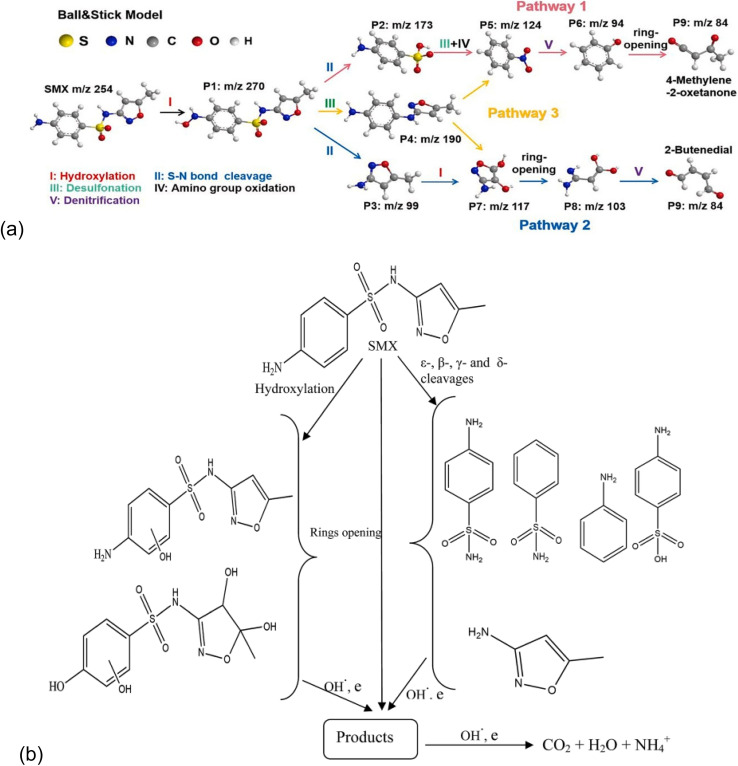
(a) Sulfamethoxazole degradation intermediates and possible reaction pathways, reproduced from ref. 71 with permission from [Elsevier],^71^ copyright 2024. (b) Proposed degradation route of sulfamethoxazole by photoelectrochemical process, reproduced from ref. 20 with permission from [RSC],^20^ copyright 2017.

As shown in Fig. 8a and b,^20,71^ five chemical reactions and degradation pathways are involved in the breakdown of sulfamethoxazole. These include hydroxylation, bond cleavage, desulfonation, oxidation, and denitrification. These reactions create intermediate products with *m*/*z* values such as 99, 94, and 84, which are lower than the molecular weight of the parent compound, with an *m*/*z* of 254.

It is important to note that, despite the interesting results obtained from various works investigating the possible chemical reactions and degradation pathways in the PEC degradation of sulfonamides, studies on the extent of toxicity of the intermediate products have not been widely reported.

## 6. Conclusion

In this report, we present the recent advancements in the photoelectrocatalytic oxidation of sulfonamides using different pristine, doped, heterojunction photoanodes, and dual photoelectrodes. Based on the summary presented in Table 2, it is evident that significant progress has been made to successfully break down sulfonamides in water. The authors which to note that in most reports in Table 2, the use of percentage degradation was in the context of the extent of parent removal rate and not the complete mineralisation rate because in degradation there is the likelihood of other intermediates in the solution. Evidently, TiO_2_ is the most widely used semiconductor photocatalyst in the PEC oxidation of sulfonamides. Despite the setbacks associated with TiO_2_, this report showed that the architecture of TiO_2_ can be tuned for improved performance. Enhancement in the PEC degradation of sulfonamides can be realised by doping and heterojunction formation. In addition, given the impressive progress made by using LED as a light source, its use is encouraged in future work as an energy-saving, cheap, and green source of light in the PEC system towards the abatement of other organic pollutants. Noticeably, Ti sheet/plate/mesh/foil and FTO glass were mostly used as the conductive substrate because Ti sheet/plate/mesh/foil could serve a dual purpose of a conductive substrate and a semiconductor amenable for doping or heterojunction formation. FTO can be a suitable substrate for different deposition techniques such as electrodeposition, successive ionic layer adsorption and reaction, simple casting, *etc.* In addition, based on the improved performance of the dual photoelectrode system using real wastewater, sunlight irradiation, and zero applied potential, this self-power generating system presents itself as a practical technology for wastewater treatment. Overall, the review shows that PEC oxidation continues to be a highly efficient technology for the abatement of organic pollutants in wastewater.

**Table 2:** Recent works on PEC oxidation of sulfonamides^a^

Photoanode	PEC condition and sulfonamide	Light source	% Degradation	Ref.
TiO_2_/Ti (UV-A)	CE: Pt plate, RE: Ag/AgCl electrode, SE: 10 mM NaCl; sulfamethoxazole	UV detector (*λ* = 267 nm)	100% within 70 min	61
BTNAs	CE: Ti mesh, RE: saturated calomel electrode, SE: 1 mM hypophosphite; sulfadiazine	Xe lamp 100 mW cm^−2^	∼80% within 2 h	73
TiO_2_-exfoliated graphite	CE: Pt foil, RE: Ag/AgCl, SE: 0.1 M Na_2_SO_4_; sulfamethoxazole	Xe lamp 100 W	∼100% after 6 h	20
Cu_0.5_Zn_0.5_Fe_2_O_4_ quantum dots/TiO_2_ NTs	CE: Pt foil, RE: saturated calomel electrode, SE: 0.1 M Na_2_SO_4_; sulfamethoxazole	Xe lamp 500 W	70% after 120 min	72
Ti/TiO_2_ NT-UiO-66 (Zr) NH_2_, Ti/TiO_2_ NT-Ru_3_(BTC)_2_, Ti/TiO_2_ NT-Au@ZIF-8	CE: Pt mesh, RE: Ag/AgCl, SE: N/A; sulfamethazine	UV light (maximum intensity of 365 nm, 12 W)	78%, 88%, 57% within 180 min	18
TiO_2_ nanoneedle arrays-Co_3_O_4_	CE: Pt foil, RE: Ag/AgCl, SE: 0.1 M Na_2_SO_4_; sulfamethazine	LED lamp 30 W	99.62% within 120 min	62
B–Bi_2_Sn_2_O_7_-OV quantum dots/Ni foam	CE: Pt foil, RE: Ag/AgCl, SE: 0.1 M Na_2_SO_4_; sulfamethazine	LED lamp 50W	100% within 60 min	19
Ag/TiO_2_/Ti	CE: Pt foil, RE: Ag/AgCl, SE: 0.1 M Na_2_SO_4_; sulfadiazine	UVA-LEDs (365 nm) and vis-LEDs (430 nm)	89.1% after 2 h	98
Ag_3_PO_4_/BiVO_4_/FTO	CE: Pt wire, RE: saturated calomel electrode; sulfamethoxazole	Xe lamp 300 W	100% within 3 h	103
Ni–Co bimetallic decorated ZIF	CE: Pt foil, RE: Ag/AgCl, SE: 0.1 M Na_2_SO_4_; sulfamethoxazole	Xe lamp 100 mW cm^−2^	100% within 24 min	2
Bi_2_Sn_2_O_7_ quantum dots/TiO_2_ nanotube arrays	CE: Pt foil, RE: Ag/AgCl, SE: 0.1 M Na_2_SO_4_; sulfamethoxazole	LED lamp 50 W	90.3% after 120 min	59
FTO–Bi_2_WO_6_	CE: Pt foil, RE: Ag/AgCl, SE: 3 mM PMS; sulfamethoxazole	Xe lamp 300 W	98% after 90 min	22
N, P co-doped black-blue TiO_2_ nanotube array	CE: N, P co-doped black-blue TiO_2_ nanotube array, RE: saturated calomel electrode, SE: 0.2 M Na_2_SO_4_; sulfamethoxazole	Xe lamp 300 W	100% after 120 min	71
FTO/BiVO_4_/NiS	CE: Pt wire, RE: Ag/AgCl, SE: 0.1 M Na_2_SO_4_; sulfamethoxazole	Xe lamp 100 W	58% within 120 min	104
g-C_3_N_4_/BiOI/EG	CE: Pt wire, RE: Ag/AgCl, SE: 0.1 M Na_2_SO_4_; sulfamethoxazole	Xe lamp 300 W	88% after 180 min	66
Co_3_Se_4_/BiVO_4_/FTO	CE: Pt wire, RE: Ag/AgCl, SE: 0.1 M Na_2_SO_4_; sulfamethoxazole	Xe lamp 100 W	75% after 120 min	17
Co-doped ZnO/FTO	CE: Ti, SE: 0.1 M Na_2_SO_4_, SE: 0.75 gr L^−1^ NaCl; sulfamethoxazole	Hg lamp 6 W	95.8% after 90 min	7
TiO_2_/Ti	CE: Pt plate, RE: Ag/AgCl, SE: 10 mM NaCl; sulfamethoxazole	UV-A lamp 4 W	100% within 70 min	61
TiO_2_/Ti	CE: stainless steel wire, RE: Ag/AgCl, SE: 0.1 M Na_2_SO_4_; sulfamethazine 98.68%	UV-A lamp 9 W	97% within 180 min	105
FTO–BiVO_4_	CE: Graphite rod, RE: Ag/AgCl, SE: 0.1 M Na_2_SO_4_; sulfamethoxazole	Xe lamp 150 W	50% within 120 min	106
FTO–Cu_2_O	CE: Pt foil, RE: Ag/AgCl, SE: 10 mM Na_2_S_2_O_8_; sulfamethoxazole	Xe lamp 100 W	86% after 120 min	21
Ti^3+^–TiO_2_NCs(N_2_)	CE: Pt sheet, RE: Ag/AgCl, SE: 0.1 M Na_2_SO_4_; sulfamethazine	LED lamp 50 W	98.68% after 60 min	54
FTO/BiVO_4_/Ag_2_S	CE: Pt sheet, RE: Ag/AgCl, SE: 0.1 M Na_2_SO_4_; sulfamethoxazole	Xe lamp 100 W	86% within 120 min	90
FTO–Cu_2_O/Ag_3_PO_4_	CE: Pt sheet, RE: Ag/AgCl, SE: 0.1 M Na_2_SO_4_; sulfamethoxazole	Xe lamp 100 W	67% after 120 min	79
FTO–AgNPs–Cu_2_O	CE: Pt sheet, RE: Ag/AgCl, SE: 3 mM Na_2_S_2_O_8_; sulfamethoxazole	Xe lamp 100 W	81% within 90 min	107
Ag_3_PO_4_/MoS_2_/TiO_2_ NTAs	CE: N/A, RE: SN/A, SE: 0.01 M Na_S_O_4_; sulfadiazine	Xe arc lamp 300 W	70% after 240 min	91

a CE = counter electrode, RE = reference electrode, SE = supporting electrolyte.

Considering the works reviewed in this article, we wish to note the following for future research:

1. About 40% of the photocatalyst used for the PEC oxidation of sulfonamides is TiO_2_. No doubt, impressive progress was made in modifying TiO_2_ in order to improve its performance. However, future works should consider the use of other highly efficient solar light-driven photocatalysts to mitigate the shortcomings of TiO_2_.

2. The discovery of different architectures of TiO_2_ such as TiO_2_ nanotube arrays, TiO_2_ nanoneedle arrays, TiO_2_ nanocone arrays, *etc.* and their performances seem to be progressive. It is not yet certain which nano architecture presents the best results; thus comparative studies can be worthwhile.

3. The engineering of S-scheme heterojunction photoanodes towards enhanced PEC oxidation of sulfonamides is still in its infancy. Thus we hope to see more research based on this architecture because of its unique internal electric field (IEF) effect which results in a more improved charge carrier separation and greater redox power which culminates to improved performance of the PEC system.

4. Defect engineering still has a lot more potential to be explored in PEC material development. For example, boron doping-mediated formation of oxygen vacancies may improve catalyst reactive sites and enhance PEC of some semiconductor photocatalysts towards the oxidation of sulfonamides and other organics in water.

5. Since the introduction of sulphate radicals into the PEC system has shown more efficiency in the degradation of sulfonamides as compared to the hydroxyl-based PEC system. We hope to see more of the sulphate radical approach in PEC degradation.

6. Synthesis of porous bimetallic/MOFs heterostructured photoanodes for PEC degradation of sulfonamides is in its infancy. Given their interesting properties and performance, more MOF-based materials should be explored to fabricate photoanodes for PEC oxidation.

7. In the PEC degradation process, there is a possibility of producing intermediates with higher toxicity than the parent compound. Thus, the need to avoid generating toxic intermediates and improve the PEC efficiency to break down toxic intermediates within a short time should be considered. Extensive studies should be carried out on toxicity.

8. Towards industrial-scale application, the dual photoelectrode system lends itself as a cheap, efficient, energy-friendly, and practical approach and should be explored.

## Author contributions

B. A. Koiki: conceptualization, methodology, writing – original draft. K. D. Jayeola: writing – review & editing. D. S. Sipuka: writing – review & editing. T. I. Sebokolodi: writing – review & editing. D. Chen: writing – review & editing. P. Liu: writing – review & editing. O. A. Arotiba: conceptualization, methodology, writing – review & editing, supervision.

## Conflicts of interest

There are no conflicts of interest to declare.

## Data Availability

No primary research results, software or code have been included and no new data were generated or analysed as part of this review.

## References

[cit1] Zainab S. M., Junaid M., Xu N., Malik R. N. (2020). Antibiotics and antibiotic resistant genes (ARGs) in groundwater: A global review on dissemination, sources, interactions, environmental and human health risks. Water Res..

[cit2] Thamilselvan A., Doong R.-A. (2023). Ni-Co bimetallic decorated dodecahedral ZIF as an efficient catalyst for photoelectrochemical degradation of sulfamethoxazole coupled with hydrogen production. Sci. Total Environ..

[cit3] Dan A., Yang Y., Dai Y.-n., Chen C.-x., Wang S.-y., Tao R. (2013). Removal and factors influencing removal of sulfonamides and trimethoprim from domestic sewage in constructed wetlands. Bioresour. Technol..

[cit4] Tolls J. (2001). Sorption of veterinary pharmaceuticals in soils: a review. Environ. Sci. Technol..

[cit5] Yang J.-F., Ying G.-G., Zhao J.-L., Tao R., Su H.-C., Liu Y.-S. (2011). Spatial and seasonal distribution of selected antibiotics in surface waters of the Pearl Rivers, China. J. Environ. Sci. Health, Part B.

[cit6] Bai Y., Ruan X., Wang F., Antoine G., van der Hoek J. P. (2019). Sulfonamides removal under different redox conditions and microbial response to sulfonamides stress during riverbank filtration: a laboratory column study. Chemosphere.

[cit7] Yeganeh M., Sobhi H. R., Behbahani M., Ghambarian M., Esrafili A. (2022). Photoelectrocatalytic degradation of sulphonamide antibiotics in aquatic media using a novel Co-doped ZnO nanocomposite: evaluation of performance, kinetic studies. Int. J. Environ. Anal. Chem..

[cit8] Mojica E.-R. and Aga D., Antibiotics Pollution in Soil and Water: Potential Ecological and Human Health Issues, 2011

[cit9] Michael I., Rizzo L., McArdell C., Manaia C., Merlin C., Schwartz T., Dagot C., Fatta-Kassinos D. (2013). Urban wastewater treatment plants as hotspots for the release of antibiotics in the environment: a review. Water Res..

[cit10] Hu X., Zhou Q., Luo Y. (2010). Occurrence and source analysis of typical veterinary antibiotics in manure, soil, vegetables and groundwater from organic vegetable bases, northern China. Environ. Pollut..

[cit11] Xu D., Xie Y., Li J. (2022). Toxic effects and molecular mechanisms of sulfamethoxazole on Scenedesmus obliquus. Ecotoxicol. Environ. Saf..

[cit12] Tačić A., Nikolić V., Nikolić L., Savić I. (2017). Antimicrobial sulfonamide drugs. Adv. Technol..

[cit13] Peng T., Song B., Wang Y., Yuan J., Yang Z., Tang L. (2025). Trophic transfer of sulfonamide antibiotics in aquatic food chains: a comprehensive review with a focus on environmental health risks. Environ. Pollut..

[cit14] Brillas E., Peralta-Hernández J. M. (2023). Removal of paracetamol (acetaminophen) by photocatalysis and photoelectrocatalysis. A critical review. Sep. Purif. Technol..

[cit15] Riaz S., Park S.-J. (2020). An overview of TiO2-based photocatalytic membrane reactors for water and wastewater treatments. J. Ind. Eng. Chem..

[cit16] Zhang D., Zhang L., An C., Wang M. (2024). Constructing Z-Scheme 3D WO3@ Co2SnO4 Heterojunction as Dual-Photocathode for Production of H2O2 and In-Situ Degradation of Organic Pollutants. Water.

[cit17] Yusuf T. L., Ogundare S. A., Opoku F., Mabuba N. (2023). Photoelectrocatalytic degradation of sulfamethoxazole over S–Scheme Co3Se4/BiVO4 heterojunction photoanode: an experimental and density functional theory investigations. Surf. Interfaces.

[cit18] Candia-Onfray C., Irikura K., Calzadilla W., Rojas S., Zanoni M. V. B., Salazar R. (2023). Degradation of contaminants of emerging concern in a secondary effluent using synthesized MOF-derived photoanodes: A comparative study between photo-, electro-and photoelectrocatalysis. Chemosphere.

[cit19] Wu H., Hu Z., Liang R., Zhang X., Zhou M., Arotiba O. A. (2023). B-doping mediated formation of oxygen vacancies in Bi2Sn2O7 quantum dots with a unique electronic structure for efficient and stable photoelectrocatalytic sulfamethazine degradation. J. Hazard. Mater..

[cit20] Peleyeju M. G., Umukoro E. H., Tshwenya L., Moutloali R., Babalola J. O., Arotiba O. A. (2017). Photoelectrocatalytic water treatment systems: degradation, kinetics and intermediate products studies of sulfamethoxazole on a TiO 2–exfoliated graphite electrode. RSC Adv..

[cit21] Koiki B. A., Orimolade B. O., Zwane B. N., Nkwachukwu O. V., Muzenda C., Ojo B. O., Nkosi D., Mabuba N., Arotiba O. A. (2021). Sulphate radical enhanced photoelectrochemical degradation of sulfamethoxazole on a fluorine doped tin oxide-copper (I) oxide photoanode. J. Electroanal. Chem..

[cit22] Orimolade B. O., Idris A. O., Feleni U., Mamba B. (2022). Peroxymonosulfate assisted photoelectrocatalytic degradation of pharmaceuticals at a FTO-Bi2WO6 electrode: Mechanism and kinetics studies. Catal. Commun..

[cit23] Tolosana-Moranchel A., McMichael S., Hamilton J., Byrne J., Fernández-Ibañez P. (2023). Electrochemically assisted photocatalytic degradation of contaminants of emerging concern in simulated wastewater using WO3–Elucidation of mechanisms. Chem. Eng. J..

[cit24] Prasannamedha G., Kumar P. S. (2020). A review on contamination and removal of sulfamethoxazole from aqueous solution using cleaner techniques: Present and future perspective. J. Clean. Prod..

[cit25] Peleyeju M. G., Arotiba O. A. (2018). Recent trend in visible-light photoelectrocatalytic systems for degradation of organic contaminants in water/wastewater. Environ. Sci.:Water Res. Technol..

[cit26] García-Galán M. J., Díaz-Cruz M. S., Barceló D. (2008). Identification and determination of metabolites and degradation products of sulfonamide antibiotics. TrAC, Trends Anal. Chem..

[cit27] Sarmah A. K., Meyer M. T., Boxall A. B. (2006). A global perspective on the use, sales, exposure pathways, occurrence, fate and effects of veterinary antibiotics (VAs) in the environment. Chemosphere.

[cit28] Duan W., Cui H., Jia X., Huang X. (2022). Occurrence and ecotoxicity of sulfonamides in the aquatic environment: A review. Sci. Total Environ..

[cit29] Ovung A., Bhattacharyya J. (2021). Sulfonamide drugs: structure, antibacterial property, toxicity, and biophysical interactions. Biophys. Rev..

[cit30] Baran W., Adamek E., Ziemiańska J., Sobczak A. (2011). Effects of the presence of sulfonamides in the environment and their influence on human health. J. Hazard. Mater..

[cit31] Dan A., Li L., Tai Y.-p., Zhang X.-m., Tao R., Yang Y. (2020). Behavior assessment of sulfonamides and N4-acetyl sulfonamides from wastewater effluent in subsurface constructed wetlands: removal, distribution, and biotransformation. Chem. Eng. J..

[cit32] Gao L., Shi Y., Li W., Niu H., Liu J., Cai Y. (2012). Occurrence of antibiotics in eight sewage treatment plants in Beijing, China. Chemosphere.

[cit33] Danner M.-C., Robertson A., Behrends V., Reiss J. (2019). Antibiotic pollution in surface fresh waters: Occurrence and effects. Sci. Total Environ..

[cit34] Luo Y., Liu C., Wang Y., Yang Y., Mishra S. (2023). Occurrence, distribution and their correlation with different parameters of antibiotics and antibiotic resistance genes in lakes of China: A review. Mar. Pollut. Bull..

[cit35] Afsa S., Hamden K., Lara Martin P. A., Mansour H. B. (2020). Occurrence of 40 pharmaceutically active compounds in hospital and
urban wastewaters and their contribution to Mahdia coastal seawater contamination. Environ. Sci. Pollut. Res..

[cit36] Ngigi A. N., Magu M. M., Muendo B. M. (2020). Occurrence of antibiotics residues in hospital wastewater, wastewater treatment plant, and in surface water in Nairobi County, Kenya. Environ. Monit. Assess..

[cit37] Lin Y.-C., Lai W. W.-P., Tung H.-h., Lin A. Y.-C. (2015). Occurrence of pharmaceuticals, hormones, and perfluorinated compounds in groundwater in Taiwan. Environ. Monit. Assess..

[cit38] Focazio M. J., Kolpin D. W., Barnes K. K., Furlong E. T., Meyer M. T., Zaugg S. D., Barber L. B., Thurman M. E. (2008). A national reconnaissance for pharmaceuticals and other organic wastewater contaminants in the United States—II) Untreated drinking water sources. Sci. Total Environ..

[cit39] Hoa P. T. P., Managaki S., Nakada N., Takada H., Shimizu A., Anh D. H., Viet P. H., Suzuki S. (2011). Antibiotic contamination and occurrence of antibiotic-resistant bacteria in aquatic environments of northern Vietnam. Sci. Total Environ..

[cit40] Yu F., Li Y., Han S., Ma J. (2016). Adsorptive removal of antibiotics from aqueous solution using carbon materials. Chemosphere.

[cit41] Nam S.-W., Choi D.-J., Kim S.-K., Her N., Zoh K.-D. (2014). Adsorption characteristics of selected hydrophilic and hydrophobic micropollutants in water using activated carbon. J. Hazard. Mater..

[cit42] Wang F., Ma S., Si Y., Dong L., Wang X., Yao J., Chen H., Yi Z., Yao W., Xing B. (2017). Interaction mechanisms of antibiotic sulfamethoxazole with various graphene-based materials and multiwall carbon nanotubes and the effect of humic acid in water. Carbon.

[cit43] de Amorim K. P., Romualdo L. L., Andrade L. S. (2013). Electrochemical degradation of sulfamethoxazole and trimethoprim at boron-doped diamond electrode: performance, kinetics and reaction pathway. Sep. Purif. Technol..

[cit44] Dias I. N., Souza B. S., Pereira J. H., Moreira F. C., Dezotti M., Boaventura R. A., Vilar V. J. (2014). Enhancement of the photo-Fenton reaction at near neutral pH through the use of ferrioxalate complexes: a case study on trimethoprim and sulfamethoxazole antibiotics removal from aqueous solutions. Chem. Eng. J..

[cit45] Pretto P. R. P., Palacio S. M., de Campos E. A., Pazini C. R., Veit M. T. (2018). Sulfamethoxazole photocatalytic degradation in a continuous flow reactor using artificial radiation. J. Environ. Chem. Eng..

[cit46] Roy K., Moholkar V. S. (2020). Sulfadiazine degradation using hybrid AOP of heterogeneous Fenton/persulfate system coupled with hydrodynamic cavitation. Chem. Eng. J..

[cit47] Qi C., Liu X., Lin C., Zhang X., Ma J., Tan H., Ye W. (2014). Degradation of sulfamethoxazole by microwave-activated persulfate: kinetics, mechanism and acute toxicity. Chem. Eng. J..

[cit48] Lin H., Niu J., Xu J., Li Y., Pan Y. (2013). Electrochemical mineralization of sulfamethoxazole by Ti/SnO2-Sb/Ce-PbO2 anode: kinetics, reaction pathways, and energy cost evolution. Electrochim. Acta.

[cit49] Martín de Vidales M. J., Robles-Molina J., Domínguez-Romero J. C., Cañizares P., Saez C., Molina-Díaz A., Rodrigo M. A. (2012). Removal of sulfamethoxazole from waters and wastewaters by conductive-diamond electrochemical oxidation. J. Chem. Technol. Biotechnol..

[cit50] Philippidis N., Pavlidou E., Sotiropoulos S., Kokkinos P., Mantzavinos D., Poulios I. (2023). Photoelectrocatalytic Oxidation of Sulfamethazine on TiO2 Electrodes. Catalysts.

[cit51] Arotiba O. A., Orimolade B. O., Koiki B. A. (2020). Visible light–driven photoelectrocatalytic semiconductor heterojunction anodes for water treatment applications. Curr. Opin. Electrochem..

[cit52] Cheng L., Jiang T., Yan K., Gong J., Zhang J. (2019). A dual-cathode photoelectrocatalysis-electroenzymatic catalysis system by coupling BiVO4 photoanode with hemin/Cu and carbon cloth cathodes for degradation of tetracycline. Electrochim. Acta.

[cit53] Annadurai T., Khedulkar A. P., Lin J.-Y., Adorna Jr J., Yu W.-J., Pandit B., Huynh T. V., Doong R.-A. (2023). S-scheme N-doped carbon dots anchored g-C3N4/Fe2O3 shell/core composite for photoelectrocatalytic trimethoprim degradation and water splitting. Appl. Catal., B.

[cit54] Liang R., Hu Z., Wu H., Li S., Zhang X., Arotiba O. A., Zhou M. (2023). Ti3+ self-doped and nitrogen-annealed TiO2 nanocone arrays photoanode for efficient visible-LED-light-driven photoelectrocatalytic degradation of sulfamethazine. Sep. Purif. Technol..

[cit55] Jayeola K. D., Sipuka D. S., Sebokolodi T. I., Nkwachukwu O. V., Muzenda C., Koiki B. A., Babalola J. O., Zhou M., Arotiba O. A. (2024). The design and characterisation of a Z-scheme Bi2O2S/ZnO heterojunction photoanode for the photoelectrochemical removal of ciprofloxacin in synthetic and real wastewater. Chem. Eng. J..

[cit56] Liu N., Lu N., Yu H., Chen S., Quan X. (2022). Enhanced degradation of organic water pollutants by photocatalytic in-situ activation of sulfate based on Z-scheme g-C3N4/BiPO4. Chem. Eng. J..

[cit57] Zou Z., Ye J., Sayama K., Arakawa H. (2001). Direct splitting of water under visible light irradiation with an oxide semiconductor photocatalyst. Nature.

[cit58] Tian J., Sang Y., Yu G., Jiang H., Mu X., Liu H. (2013). A Bi2WO6-based hybrid photocatalyst with broad spectrum photocatalytic properties under UV, visible, and near-infrared irradiation. Adv. Mater..

[cit59] Wu H., Hu Z., Liang R., Nkwachukwu O. V., Arotiba O. A., Zhou M. (2023). Novel Bi2Sn2O7 quantum dots/TiO2 nanotube arrays S-scheme heterojunction for enhanced photoelectrocatalytic degradation of sulfamethazine. Appl. Catal., B.

[cit60] Nkwachukwu O. V., Muzenda C., Koiki B. A., Arotiba O. A. (2023). Perovskites in photoelectrocatalytic water treatment: Bismuth ferrite - graphite nanoparticles composite photoanode for the removal of ciprofloxacin in water. J. Photochem. Photobiol., A.

[cit61] Su Y.-f., Wang G.-B., Kuo D. T. F., Chang M.-l., Shih Y.-h. (2016). Photoelectrocatalytic degradation of the antibiotic sulfamethoxazole using TiO2/Ti photoanode. Appl. Catal., B.

[cit62] Hu Z., Liang R., Song X., Wu H., Sun J., Liu J., Zhou M., Arotiba O. A. (2023). Efficient Bias-Free Degradation of Sulfamethazine by TiO2 Nanoneedle Arrays Photoanode and Co3O4 Photocathode System under LED-Light Irradiation. Catalysts.

[cit63] Ntsendwana B., Sampath S., Mamba B. B., Arotiba O. A. (2013). Photoelectrochemical oxidation of p-nitrophenol on an expanded graphite—TiO2 electrode. Photochem. Photobiol. Sci..

[cit64] Ntsendwana B., Mamba B. B., Sampath S., Arotiba O. A. (2013). Synthesis, characterisation and application of an exfoliated graphite–diamond composite electrode in the electrochemical degradation of trichloroethylene. RSC Adv..

[cit65] Ama O. M., Mabuba N., Arotiba O. A. (2015). Synthesis, Characterization, and Application of Exfoliated Graphite/Zirconium Nanocomposite Electrode for the Photoelectrochemical
Degradation of Organic Dye in Water. Electrocatalysis.

[cit66] Mafa P. J., Kuvarega A. T., Mamba B. B., Ntsendwana B. (2019). Photoelectrocatalytic degradation of sulfamethoxazole on g-C3N4/BiOI/EG pn heterojunction photoanode under visible light irradiation. Appl. Surf. Sci..

[cit67] Mahhumane N., Cele L. M., Muzenda C., Nkwachukwu O. V., Orimolade B. O., Koiki B. A., Tshwenya L., Arotiba O. A. (2022). Photoelectrocatalytic application of a superhydrophilic exfoliated graphite supported iron doped Bi2WO6 photoanode for the degradation of Orange II dye in water. Mater. Today Commun..

[cit68] Aouaj M. A., Diaz R., Belayachi A., Rueda F., Abd-Lefdil M. (2009). Comparative study of ITO and FTO thin films grown by spray pyrolysis. Mater. Res. Bull..

[cit69] Orimolade B. O., Zwane B. N., Koiki B. A., Tshwenya L., Peleyeju G. M., Mabuba N., Zhou M., Arotiba O. A. (2020). Solar photoelectrocatalytic degradation of ciprofloxacin at a FTO/BiVO4/MnO2 anode: kinetics, intermediate products and degradation pathway studies. J. Environ. Chem. Eng..

[cit70] Chemelewski W. D., Mabayoje O., Mullins C. B. (2015). SILAR growth of Ag3VO4 and characterization for photoelectrochemical water oxidation. J. Phys. Chem. C.

[cit71] Li S., Zhang G., Meng D., Yang F. (2024). Photoelectrocatalytic activation of sulfate for sulfamethoxazole degradation and simultaneous H2 production by bifunctional N, P co-doped black-blue TiO2 nanotube array electrode. Chem. Eng. J..

[cit72] Fan S., Li X., Zhao Q., Zeng L., Zhang M., Yin Z., Lian T., Tadé M. O., Liu S. (2018). Rational design and synthesis of highly oriented copper–zinc ferrite QDs/titania NAE nano-heterojunction composites with novel photoelectrochemical and photoelectrocatalytic behaviors. Dalton Trans..

[cit73] Meng D., Zhang G., Li S., Yang F. (2024). Photoelectrochemical intensified efficient phosphorus recovery from hypophosphite and sulfadiazine co-contaminated wastewater by blue TiO2 nanotube arrays anode. Sep. Purif. Technol..

[cit74] Koiki B. A., Orimolade B. O., Zwane B. N., Nkosi D., Mabuba N., Arotiba O. A. (2020). Cu2O on anodised TiO2 nanotube arrays: A heterojunction photoanode for visible light assisted electrochemical degradation of pharmaceuticals in water. Electrochim. Acta.

[cit75] Lei X., Wang B., Liu J., Ye Z., Chang Z., Jiang M., Sun X. (2014). Three-dimensional NiAl-mixed metal oxide film: preparation and capacitive deionization performances. RSC Adv..

[cit76] Wang Y.-L., Zhao Y.-Q., Xu C.-L., Zhao D.-D., Xu M.-W., Su Z.-X., Li H.-L. (2010). Improved performance of Pd electrocatalyst supported on three-dimensional nickel foam for direct ethanol fuel cells. J. Power Sources.

[cit77] Yuan J., Hu H., Chen M., Shi J., Shangguan W. (2008). Promotion effect of Al2O3–SiO2 interlayer and Pt loading on TiO2/nickel-foam photocatalyst for degrading gaseous acetaldehyde. Catal. Today.

[cit78] Wang M., Xu Z., Wang J., Kang J., Tang Y., Ma T., Dong Q. (2023). Cu doped Fe2O3 growing a nickel foam for sulfadiazine degradation in peroxymonosulfate assisting photo-electrochemical system: Performance, mechanism and degradation pathway. Chem. Eng. J..

[cit79] Koiki B. A., Orimolade B. O., Zwane B. N., Nkwachukwu O. V., Muzenda C., Nkosi D., Arotiba O. A. (2021). The application of FTO-Cu2O/Ag3PO4 heterojunction in the photoelectrochemical degradation of emerging pharmaceutical pollutant under visible light irradiation. Chemosphere.

[cit80] Koiki B. A., Muzenda C., Jayeola K. D., Zhou M., Marken F., Arotiba O. A. (2023). Sulfate Radical in (Photo) electrochemical Advanced Oxidation Processes for Water Treatment: A Versatile Approach. J. Phys. Chem. Lett..

[cit81] Chen X., Liu L., Huang F. (2015). Black titanium dioxide (TiO2) nanomaterials. Chem. Soc. Rev..

[cit82] Jia M., Yang Z., Xiong W., Cao J., Xiang Y., Peng H., Jing Y., Zhang C., Xu H., Song P. (2021). Magnetic heterojunction of oxygen-deficient Ti3+-TiO2 and Ar-Fe2O3 derived from metal-organic frameworks for efficient peroxydisulfate (PDS) photo-activation. Appl. Catal., B.

[cit83] Yang D., Liang J., Luo L., Deng R., Li G., He Q., Chen Y. (2021). Facile defect engineering in ZnIn2S4 coupled with carbon dots for rapid diclofenac degradation. Chin. Chem. Lett..

[cit84] Liu Z., Zhao Z., Wang Y., Dou S., Yan D., Liu D., Xia Z., Wang S. (2017). In situ exfoliated, edge-rich, oxygen-functionalized graphene from carbon fibers for oxygen electrocatalysis. Adv. Mater..

[cit85] Xiao Z., Wang Y., Huang Y.-C., Wei Z., Dong C.-L., Ma J., Shen S., Li Y., Wang S. (2017). Filling the oxygen vacancies in Co 3 O 4 with phosphorus: an ultra-efficient electrocatalyst for overall water splitting. Energy Environ. Sci..

[cit86] Yan D., Li Y., Huo J., Chen R., Dai L., Wang S. (2017). Defect chemistry of nonprecious-metal electrocatalysts for oxygen reactions. Adv. Mater..

[cit87] Xu W., Lyu F., Bai Y., Gao A., Feng J., Cai Z., Yin Y. (2018). Porous cobalt oxide nanoplates enriched with oxygen vacancies for oxygen evolution reaction. Nano Energy.

[cit88] Zhuang L., Ge L., Yang Y., Li M., Jia Y., Yao X., Zhu Z. (2017). Ultrathin iron-cobalt oxide nanosheets with abundant oxygen vacancies for the oxygen evolution reaction. Adv. Mater..

[cit89] Jia M., Xiong W., Yang Z., Cao J., Zhang Y., Xiang Y., Xu H., Song P., Xu Z. (2021). Metal-organic frameworks and their derivatives-modified photoelectrodes for photoelectrochemical applications. Coord. Chem. Rev..

[cit90] Orimolade B. O., Arotiba O. A. (2020). Towards visible light driven photoelectrocatalysis for water treatment: application of a FTO/BiVO4/Ag2S heterojunction anode for the removal of emerging pharmaceutical pollutants. Sci. Rep..

[cit91] Teng W., Xu J., Cui Y., Yu J. (2020). Photoelectrocatalytic degradation of sulfadiazine by Ag3PO4/MoS2/TiO2 nanotube array electrode under visible light irradiation. J. Electroanal. Chem..

[cit92] Wang C.-y., Pagel R., Dohrmann J. K., Bahnemann D. W. (2006). Antenna mechanism and deaggregation concept: novel mechanistic principles for photocatalysis. C. R. Chim..

[cit93] Guo R.-t., Guo S.-h., Yu L.-q., Pan W.-g. (2023). Recent Progress and Perspectives of S-Scheme Heterojunction Photocatalysts for Photocatalytic CO2 Reduction. Energy Fuels.

[cit94] Toe C. Y., Scott J., Amal R., Ng Y. H. (2019). Recent advances in suppressing the photocorrosion of cuprous oxide for photocatalytic and photoelectrochemical energy conversion. J. Photochem. Photobiol., C.

[cit95] Chen Q., Li J., Li X., Huang K., Zhou B., Cai W., Shangguan W. (2012). Visible-light responsive photocatalytic fuel cell based on WO3/W photoanode and Cu2O/Cu photocathode for simultaneous wastewater treatment and electricity generation. Environ. Sci. Technol..

[cit96] Yang W., Prabhakar R. R., Tan J., Tilley S. D., Moon J. (2019). Strategies for enhancing the photocurrent, photovoltage, and stability of photoelectrodes for photoelectrochemical water splitting. Chem. Soc. Rev..

[cit97] Hu Z., Zhou M., Maitlo H. A., Liang R., Zheng Y., Wu H., Song X., Arotiba O. A. (2023). Novel dual-photoelectrode photoelectrocatalytic system based on TiO2 nanoneedle arrays photoanode and nitrogen-doped carbon dots/Co3O4 photocathode for efficient water purification at low/no applied voltage. Appl. Catal., B.

[cit98] Leng H., Li Z., Li W., Lv Z., Guo J., You H., Jia Y., Zhang G., Wang L. (2024). Synergy of dual photoelectrodes for simultaneous antibiotic degradation and CO2 reduction by Z-scheme PEC system. Sep. Purif. Technol..

[cit99] Fan Y., Ji Y., Kong D., Lu J., Zhou Q. (2015). Kinetic and mechanistic investigations of the degradation of sulfamethazine in heat-activated persulfate oxidation process. J. Hazard. Mater..

[cit100] Wan Z., Wang J. (2017). Degradation of sulfamethazine using Fe3O4-Mn3O4/reduced graphene oxide hybrid as Fenton-like catalyst. J. Hazard. Mater..

[cit101] Wang A., Chen Y., Zheng Z., Wang H., Li X., Yang Z., Qiu R., Yan K. (2021). In situ N-doped carbon-coated mulberry-like cobalt manganese oxide boosting for visible light driving photocatalytic degradation of pharmaceutical pollutants. Chem. Eng. J..

[cit102] Du X., Fu W., Su P., Cai J., Zhou M. (2020). Internal-micro-electrolysis-enhanced heterogeneous electro-Fenton process catalyzed by Fe/Fe3C@ PC core–shell hybrid for sulfamethazine degradation. Chem. Eng. J..

[cit103] Cao D., Wang Y., Qiao M., Zhao X. (2018). Enhanced photoelectrocatalytic degradation of norfloxacin by an Ag3PO4/BiVO4 electrode with low bias. J. Catal..

[cit104] Mohlala T., Yusuf T. L., Mabuba N. (2023). Photoelectrocatalytic degradation of emerging organic pollutants in water on an FTO/BiVO4/NiS photoanode. J. Electroanal. Chem..

[cit105] Philippidis N., Pavlidou E., Sotiropoulos S., Kokkinos P., Mantzavinos D., Poulios I. (2023). Photoelectrocatalytic Oxidation of Sulfamethazine on TiO2 Electrodes. Catalysts.

[cit106] Fuentes-Camargo I., Carrera-Crespo J. E., Vazquez-Arenas J., Romero-Ibarra I., Rodríguez J. L., Lartundo-Rojas L., Cardoso-Martínez J. (2019). Pulse-plating electrodeposition of metallic Bi in an organic-free aqueous electrolyte and its conversion into BiVO4 to improve photoelectrochemical activity toward pollutant degradation under visible light. J. Phys. Chem. C.

[cit107] Koiki B. A., Arotiba O. A. (2024). Persulphate assisted photoelectrochemical degradation of organic pollutants in water on a silver nanoparticle modified Cu2O photoanode. J. Electroanal. Chem..

